# Impact of Learning Motivation and Presentation Modalities on Cognitive Load and Learning Performance in Preoperative Digital Health Education for Older Patients With Knee Arthroplasty: Psychobehavioral Experimental Study

**DOI:** 10.2196/79430

**Published:** 2025-10-24

**Authors:** Yawen Li, Chuchu Yan, Ru Chen, Haiying Lu, Yawei Shan

**Affiliations:** 1 School of Nursing, Shanghai University of Traditional Chinese Medicine, No. 1200 Cailun Road, Shanghai, 201203, China, +86-021-51323095; 2 Department of Nursing, Shanghai Guanghua Hospital of Integrated Traditional Chinese and Western Medicine, Shanghai, China

**Keywords:** cognitive load theory, digital health education, eye-tracking, knee arthroplasty, learning motivation

## Abstract

**Background:**

Effective preoperative digital health education (DHE) is critical for patients undergoing knee arthroplasty (KA), particularly older adults who face age-related cognitive vulnerabilities. Cognitive Load Theory suggests that presentation modality and learning motivation strongly influence cognitive processing, yet their combined effects in clinical education remain underexplored.

**Objective:**

This study investigated how learning motivation and presentation modality (text-only, text-graphic composite, and video-based) affect cognitive load and learning performance in older adult patients preparing for KA.

**Methods:**

A 2×3 factorial psychobehavioral experiment was conducted with 62 patients (≥60 y) stratified by learning motivation (high vs low). Each participant completed 6 DHE modules delivered across the 3 modalities. Cognitive load was measured using subjective ratings (Chinese National Aeronautics and Space Administration Task Load Index) and objective eye-tracking indicators (average fixation duration, number of fixations, and time to first fixation). Learning performance was assessed through knowledge retention and transfer tests (30 items). Data were analyzed using general linear models, analysis of covariance, and correlation analyses, with covariates including knee function, prior knowledge, eHealth literacy, and psychological distress.

**Results:**

High-motivation learners demonstrated significantly lower cognitive load (National Aeronautics and Space Administration Task Load Index; large effect size) and superior learning performance (medium effect size) compared to low-motivation learners. Video-based materials consistently yielded the lowest extraneous load and supported efficient knowledge acquisition (medium effect size). Text-graphic composites elicited higher cognitive load but facilitated deeper processing and schema construction, particularly for highly motivated learners (medium-to-large effect sizes). Eye tracking confirmed these dynamics: high-motivation participants showed shorter fixation durations and more efficient allocation of attention across modalities, whereas low-motivation learners displayed scattered fixation patterns, especially in text-graphic conditions. Across the sample, cognitive load was negatively correlated with learning performance (large effect size), reinforcing its role as a key mediator of digital learning outcomes.

**Conclusions:**

Both learning motivation and modality exert significant, though partly independent, influences on preoperative DHE outcomes in patients with KA. Video-based content enhances cognitive efficiency, while text-graphic formats may promote germane load among motivated learners. These findings highlight the importance of motivational scaffolding, adaptive modality selection, and the integration of real-time cognitive monitoring in DHE design. Beyond informing digital education for KA, the study demonstrates the feasibility of applying psychobehavioral experimental methods with older surgical patients, offering a framework for optimizing cognitive alignment in clinical education.

## Introduction

Knee arthroplasty (KA) is a widely accepted surgical intervention for individuals with end-stage knee osteoarthritis and rheumatoid arthritis, offering substantial relief from pain and improved joint function [1]. Despite these clinical benefits, approximately 20% of patients report dissatisfaction with postoperative outcomes [2], largely due to poor adherence to rehabilitation protocols, misconceptions about expected recovery, and insufficient preoperative preparation [3]. Preoperative health education has proven effective in improving patient comprehension of the surgical procedure, postoperative expectations, and rehabilitation protocols, thereby enhancing recovery adherence and clinical outcomes [4,5].

With the proliferation of digital technology, digital health education (DHE) has emerged as a widely adopted modality, providing structured, customizable, and repeatable learning experiences [4]. Such tools provide scalable solutions, particularly for elective surgical pathways like KA. However, DHE for this patient group presents 2 pressing challenges: (1) the lack of standardized content, leading to inconsistencies in material quality, readability, and scientific validity [6], and (2) the unique cognitive vulnerabilities of older adult learners, who often face age-related declines in working memory, attentional control, and digital literacy [7]. These factors heighten the risk of cognitive overload, thereby impairing learning outcomes.

To address these challenges, the effects of 2 key independent variables on digital learning performance should be investigated: learning motivation and presentation modality (text, text-graphic composite, and video-based). Cognitive Load Theory (CLT) provides a robust framework for understanding how these variables influence cognitive processes and learning outcomes [8]. CLT emphasizes the limited capacity of working memory and posits that instructional design should manage 3 types of cognitive load (Figure S1 in Multimedia Appendix 1): intrinsic (task complexity), extraneous (presentation format), and germane (learner-driven effort for schema construction). Paas et al further elaborated this model by distinguishing between causal and measurement dimensions [9] (Figure S2 in Multimedia Appendix 1). Therefore, effective digital instructional design must manage intrinsic load, minimize extraneous load, and optimize germane load to maximize learning outcomes.

Several CLT principles are particularly relevant to DHE. The modality effect demonstrates that distributing information across visual and auditory channels (eg, narration with diagrams) reduces extraneous load and facilitates comprehension [10]. The segmentation effect highlights that breaking complex materials into smaller, learner-paced segments improves retention and reduces overload [11]. The transient information effect emphasizes that rapidly disappearing information (eg, continuous video streams) can overwhelm working memory unless supplemented by cues, scaffolds, or learner control [12]. Accordingly, presentation modality is conceptualized as a key determinant of extraneous cognitive load, while learning motivation contributes to germane load by fostering deeper engagement and schema development. Situating the intervention groups within these CLT effects allows this study to move beyond simple media comparison, offering a theoretically grounded investigation into how instructional design and learner characteristics jointly shape cognitive load and digital learning performance.

Prior research has demonstrated that presentation modality significantly influences information processing, particularly in older adults [13,14]. Grounded in multimedia learning theory, instructional strategies that integrate multiple sensory modalities, such as combining video with narration, can optimize cognitive channel allocation, reduce extraneous load, and enhance learning efficiency [10,15]. In clinical education contexts, video-based formats have been shown to perform particularly well due to their capacity for dynamic visual demonstration, minimizing cognitive conflict, and facilitating deeper information integration [13]. However, alternative evidence suggests that static, text-graphic composites may be more suitable for older adults, as these formats reduce reading burden and enhance information comprehensibility. By presenting concise text alongside images, illustrated materials enable older adult learners to intuitively access content while reducing cognitive effort [14]. These findings underscore the importance of tailoring presentation modality to the cognitive and perceptual preferences of older learners, particularly in preoperative educational contexts.

In parallel, learning motivation plays a critical role as a driver of germane cognitive load. High motivation facilitates sustained cognitive engagement, improves comprehension, and positively influences both digital learning performance and technology use intention [16]. Despite its importance, few studies have examined the impact of learning motivation on cognitive load and educational effectiveness among older adult patients preparing for KA [6]. This gap is especially relevant given that cognitive load in this population may also be shaped by other psychological and contextual variables. In this study, factors including knee function, prior knowledge, eHealth literacy, psychological distress, technophobia, digital technology support, and self-efficacy are treated as covariates. Their relevance has been previously validated by research [5], and they are included here to ensure a more accurate estimation of the unique effects of presentation modality and learning motivation.

To empirically examine these relationships, this study adopts a psychobehavioral experimental design incorporating both subjective and objective indicators of cognitive load. While subjective assessments such as the National Aeronautics and Space Administration Task Load Index (NASA-TLX) are widely used, they are inherently limited in capturing the dynamic and real-time nature of cognitive processing [17]. Eye-tracking technology offers a robust alternative, enabling continuous, high-resolution measurement of attention and cognitive effort through indicators such as fixation duration, first fixation latency, and heatmaps. These metrics provide valuable insight into learners’ visual attention allocation and processing strategies, especially among older adults, who often exhibit narrowed attentional breadth and increased refixation frequency, both markers of cognitive inefficiency [18]. When integrated within a psychobehavioral experimental framework, eye-tracking allows for nuanced analysis of how presentation modalities and learner motivation interact to influence cognitive load, thereby informing the design of more cognitively aligned DHE materials.

**Figure 1.**
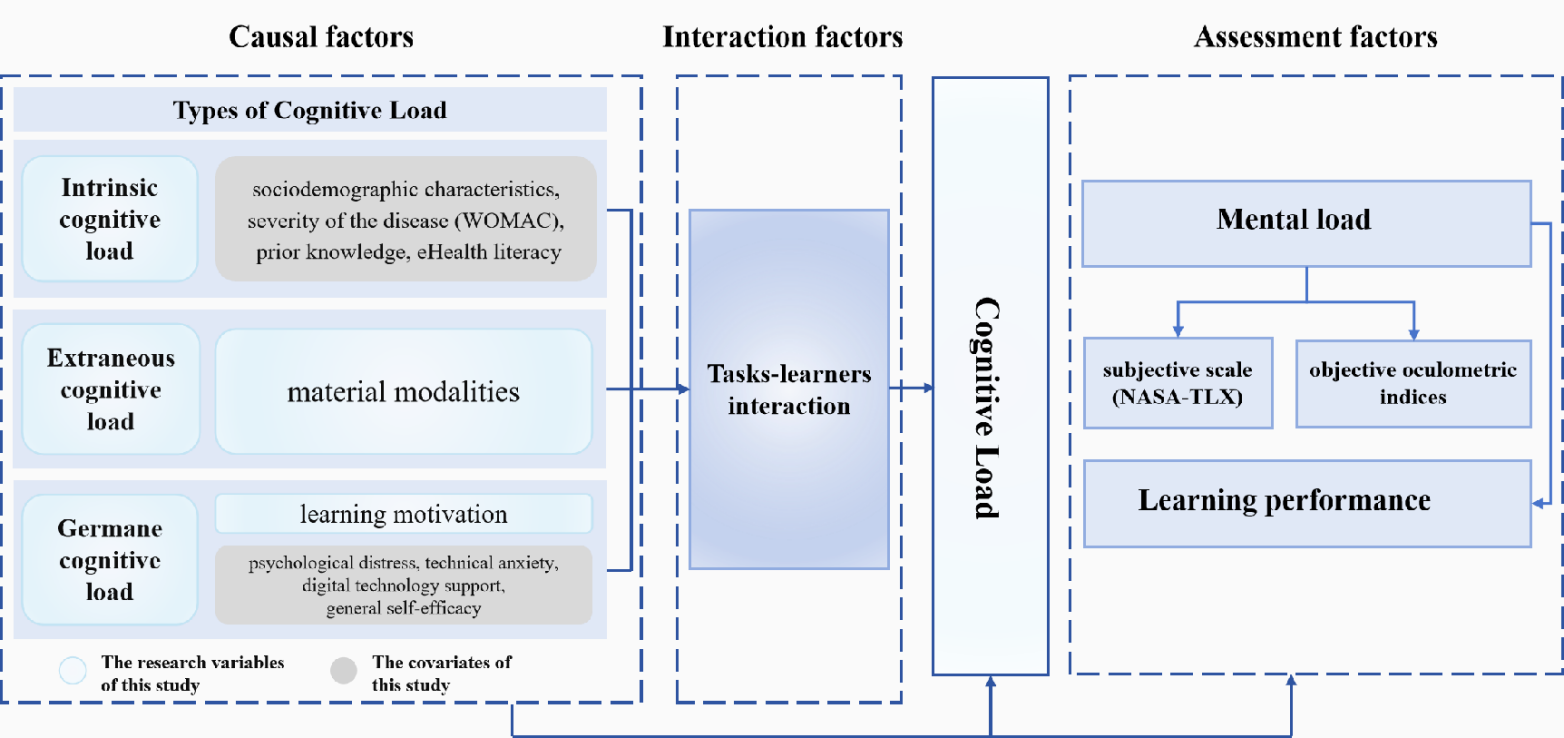
Theoretical framework of the study investigating the impact of presentation modality and learning motivation on cognitive load and learning performance in preoperative digital health education among patients with knee arthroplasty (Shanghai, China; experimental study conducted from July to December 2024). NASA-TLX: National Aeronautics and Space Administration Task Load Index; WOMAC: Western Ontario and McMaster Universities Osteoarthritis Index.

Based on the above theoretical and methodological framework (integrating CLT and 2D structural model of cognitive load—Figure 1), this study tests the following hypotheses:

- H1: Presentation modality (inducing extraneous load) and learning motivation (fostering germane load) significantly affect cognitive load and learning performance, through both main and interaction effects.
- H2: Cognitive load modulates learning performance by influencing attentional allocation patterns.

By adopting CLT as the guiding theory and utilizing psychobehavioral methods, this study aims to identify key instructional and learner-related determinants of effective DHE for elderly surgical patients, ultimately contributing to the development of more personalized and cognitively aligned educational strategies.

## Methods

### Design

This study employed a 2×3 factorial psychobehavioral experimental design. The 2 between-subject factors were learning motivation (high vs low) and presentation modality (text, text-graphic composite, and video-based). Covariates included knee function, prior knowledge, eHealth literacy, psychological distress, technophobia, and self-efficacy. Participants were stratified by learning motivation and completed standardized pretests to measure covariates. The primary outcomes were cognitive load (assessed subjectively and via eye-tracking) and learning performance (knowledge retention and transfer).

To control for potential order effects across different modules, a 6×6 Latin square design was employed. Each participant learned 6 distinct modules (eg, anatomy, procedure, rehabilitation), with 2 delivered in text-only format, 2 in text-graphic composite format, and 2 in video-based format. No content was repeated across formats. The Latin square counterbalanced both the sequence of modules and the modality assignments such that each modality appeared equally often in each ordinal position. Participants were randomly assigned to one of the 6 Latin square rows, ensuring systematic rotation and balanced exposure across the sample.

### Participants and Sample

Participants were recruited through purposive sampling from the joint surgery ward of a tertiary hospital in Shanghai, China. The recruitment period spanned from July 1, 2024, to December 25, 2024. Potential participants were initially identified by the clinical nursing staff based on preoperative admission records. A member of the research team then approached eligible patients in person at their bedside 1 day prior to surgery. Each potential participant received a detailed explanation of the study’s purpose, procedures, potential risks and benefits, and their rights, including the right to withdraw at any time without affecting their medical care. Written informed consent was obtained from all individuals who agreed to participate.

The inclusion criteria included (1) age ≥60 years, (2) diagnosis of primary knee osteoarthritis or rheumatoid arthritis and scheduled for knee arthroplasty; (3) corrected visual acuity ≥1.0 and normal hearing (≤25 dB), (4) adequate comprehension or verbal communication ability, (5) provision of informed consent, and (6) classified learning motivation (low ≤24, high ≥51).

The exclusion criteria included (1) severe comorbidities (eg, coronary artery disease, chronic obstructive pulmonary disease, major cerebrovascular disease), (2) enrollment in concurrent DHE studies, and (3) inability to complete 5-point eye-tracker calibration. Participants were excluded from the final analysis if they had missing data on key variables or an effective eye-tracking sampling rate below 60%.

A priori power analysis in G*Power 3.1 for a mixed ANOVA targeting the within-between interaction was performed. We assumed a medium effect size of *f*=0.25 (equivalent to partial *η*² = 0.059), *α*=.05, 1-*β*=.80, 2 groups (high vs low motivation), 3 measurements (modality levels), correlation among repeated measures=0.50 (from pilot data), and nonsphericity correction *ε*=1.0. Under these assumptions, the required total sample was 56 (28 per motivation group). As a sensitivity check, using a more conservative *ε*=0.75 or a lower within-person correlation (*r*=0.30) increases the requirement to N = 62‐70. Anticipating up to 5% attrition, we targeted N=66. Our achieved sample (N=66, with 33 per group) met this target.

### Learning Materials

Learning materials were systematically developed to cover essential perioperative education for KA, including 6 modules: (1) knee anatomy, (2) procedure overview, (3) implant selection, (4) surgical process, (5) rehabilitation exercises, and (6) expected outcomes (File S1 in Multimedia Appendix 1). Content development was grounded in evidence-based medicine. A systematic search of clinical guideline repositories (eg, AHRQ, AAOS, NICE, OARSI, and G-I-N) and bibliographic databases (Cochrane Library, PubMed, Embase, Web of Science, PsycINFO) was conducted up to September 2022. Educational topics were identified, appraised for quality, and synthesized to construct the draft materials.

To ensure clinical accuracy and practical relevance, content was refined through expert consensus involving 10 interdisciplinary professionals (surgeons, nurses, anesthetists, physical therapists, and dietitians), including individuals with lived experience advising on patient needs. Given the cognitive vulnerabilities of older adult patients and preoperative psychological stress, the length, complexity, and readability of materials were strictly controlled to prevent cognitive overload, which could otherwise hinder the experiment. Revisions incorporated feedback on clarity, comprehensiveness, and patient-centeredness. Readability was assessed using the Chinese Reading Ease and Grade Level Readability Tests (targeting grade-4 level) and the Criteria of Importance via Intercriteria Correlation [19], ensuring accessibility for older adult learners. Materials were further evaluated with the Patient Education Materials Assessment Tool [20].

Instructional design followed Mayer’s Cognitive Theory of Multimedia Learning [21], integrating CLT principles. The text-only format served as a nonmultimedia control (single-channel visual-verbal). The text-graphic composite represented static multimedia, employing the segmentation effect by chunking content into short units to support integration of text and visuals [11]. The video-based format represented dynamic multimedia, leveraging the modality effect (synchronized audio-visual delivery) while incorporating learner control (play/pause, subtitles) to mitigate the transient information effect [12].

Across all formats, the learning materials were standardized in typography, layout, and timing. Each module was independent in content, with difficulty carefully equalized and designed to require approximately 3‐5 minutes to complete. To ensure comparability, the same core information was conveyed across text, text-graphic, and video formats. In the text-graphic condition, graphics illustrated the corresponding textual information without adding new concepts, while in the video condition, narration and visual demonstrations followed the same script and sequence as the text version. This ensured consistency in length, content coverage, and instructional emphasis across all formats.

Prior to the full-scale experiment, an observational study involving 202 older patients with KA (≥60 y) evaluated the digital platform-based materials. Based on their feedback, revisions were made to improve readability, clarity, and engagement. Subsequently, a pilot test with 50 patients was conducted to further refine the content and format. These preparatory steps confirmed both the feasibility of the materials and the importance of tailoring digital health education to the cognitive profiles of older adults.

### Measures and Data Collection

Baseline assessments included sociodemographic and clinical characteristics, as well as key covariates: knee function, prior knowledge, eHealth literacy, psychological distress, technophobia, digital technology support, and self-efficacy. After completing each DHE module, participants’ subjective cognitive load was measured. Upon completion of all modules, learning performance was assessed via knowledge tests. All procedures were conducted under conditions of voluntary participation with continuous informed consent.

### Sociodemographic and Clinical Characteristics

Demographic and clinical data were collected using a self-designed questionnaire (see File S2 in Multimedia Appendix 1). Variables included age, gender, residential setting, educational attainment, marital status, parental status, health insurance coverage, occupational category, and household income. Clinical information included the primary diagnosis, disease duration, planned surgical procedure, and comorbid conditions. All income data were reported in CNY. For reference, the average exchange rate at the time of the study was 1 US $ ≈7.19 CNY.

### Learning Motivation

Learning motivation in the mobile environment was assessed using the Chinese-adapted Mobile Learning Motivation Scale [22]. This 13-item instrument employs a 5-point Likert scale (1=strongly disagree to 5=strongly agree). The Cronbach *α* coefficient of this scale in this study was .941. Motivation classification thresholds were established through prior research: in a cross-sectional survey of 202 patients before KA surgery [5], scores were rank-ordered and extreme group analysis (top/bottom 27%) revealed cutoff values. Participants scoring ≤24 (bottom 27%) were classified as low motivation, while those scoring ≥51 (top 27%) were classified as high motivation.

### Cognitive Load

The measurement of cognitive load includes subjective measurement and objective measurement. The subjective measurement is evaluated using the NASA-TLX, which consists of 6 dimensions: mental demand, physical demand, temporal demand, performance, effort, and frustration [17]. Each dimension is represented by a 20-point linear scale ranging from 0 to 100. The higher the score, the higher the cognitive load. The total score is the average of the scores of each dimension. In this study, the Cronbach *α* coefficient of this scale is .843.

Eye movement data were collected using a Tobii Pro screen–based eye tracker to capture objective indicators of cognitive load during task performance. At least 3 primary metrics were analyzed: average fixation duration, number of fixation points, and duration before the first fixation. Average fixation duration reflects the depth of cognitive processing, with longer fixations indicating greater effort in integrating or resolving information, particularly when processing complex multimedia stimuli. The number of fixation points provides an estimate of attentional allocation, where higher counts may signal increased visual search or divided attention, suggesting greater extraneous load when learners must navigate multiple information sources. Duration before the first fixation captures the efficiency of initial attentional orientation, with shorter times reflecting faster access to relevant information, whereas longer delays may suggest difficulties in identifying salient elements, an issue especially pertinent to older adults facing age-related declines in visual scanning efficiency. Together, these indicators offer an objective complement to subjective ratings, enabling a nuanced assessment of how multimedia design influences intrinsic and extraneous cognitive load in preoperative patient education.

### Learning Performance

Learning performance refers to the measurable outcome of knowledge acquisition and skill development [23,24]. In this study, learning performance is defined as the immediate educational outcome, measured through participants’ ability to both retain information and transfer knowledge to solve novel problems. Learning performance was assessed using a structured examination aligned with the 6 digital education modules (File S3 in Multimedia Appendix 1). Each module was followed by 5 single-choice questions, resulting in a total of 30 items of the examination. Items were developed according to predefined learning objectives, comprising 3 recall-based questions and 2 transfer-based questions per module. Module-level performance was quantified as the mean module score (range: 0‐5 points), calculated as the total score achieved within a single module. To derive an aggregate measure of overall performance, the overall mean module score was computed for each participant by summing scores across all 6 modules and dividing by 6 (range: 0‐5 points). This approach accounted for potential intermodule variability while providing a composite metric of knowledge retention.

Content validity was ensured through expert consensus with a panel comprising 2 associate professors of surgical nursing, 1 surgeon, 1 registered nurse, and 1 clinical kinesiologist. A pilot test was conducted with 50 patients. In this study, item difficulty and discrimination indices were calibrated, yielding mean item difficulty values of 0.512‐0.570 and item discrimination values of 0.412‐0.486. For transparency, internal consistency was also examined. The Kuder-Richardson 20 (KR-20) coefficient for the full 30-item test was 0.498, whereas module-level KR-20 values averaged ≈0.250. These modest values are attributable to the short length of each module subtest (5 items), the intentional sampling of heterogeneous content domains, and the inclusion of both recall and transfer items with differing difficulty levels. Given these design characteristics and the clinical need to minimize participant burden, KR-20 was not considered an informative indicator of test quality in this context. Instead, content validity, item difficulty, and discrimination indices were prioritized to ensure that the instrument adequately sampled the learning objectives and captured knowledge acquisition in preoperative older adult patients.

### Covariates

#### Knee Function

Knee function was measured using the Western Ontario and McMaster Universities Osteoarthritis Index, a validated 24-item instrument covering pain, stiffness, and physical function on a 5-point Likert scale [25]. Higher scores indicate more severe symptoms. In this study, the Cronbach *α* was .843.

#### Prior Knowledge

A self-developed 6-item scale was used to assess prior knowledge related to health education, including previous exposure to health education, digital learning experience, and competence in using digital tools (see File S4 in Multimedia Appendix 1). Responses were recorded on a 10-point Likert scale (1=no experience and 10=expert-level competence). The Cronbach *α* was .815.

#### eHealth Literacy

The Chinese version of the eHealth Literacy Scale was used to assess participants’ ability to locate, evaluate, and apply online health information [26,27]. The scale includes 8 items rated on a 5-point Likert scale. Total scores range from 8 to 40, with scores ≥32 indicating adequate literacy. The Cronbach *α* in this study was .771.

#### Psychological Distress

Psychological distress was assessed using the Kessler Psychological Distress Scale (K10), which measures the frequency of emotional distress symptoms over the past 30 days across 10 items [28]. Each item is rated from 1 (never) to 5 (always), yielding a total score from 10 to 50. Higher scores indicate more severe distress. The Cronbach *α* in this study was .756.

#### Technophobia

Technological anxiety was evaluated using a 13-item Technophobia Scale adapted from Khasawneh and revised by Sun [29]. The scale assesses technological tension, fear, and privacy concerns using a 5-point Likert scale (1=completely inconsistent and 5=completely consistent). Higher scores reflect greater technophobia. The Cronbach *α* was .850.

#### Self-Efficacy

Self-efficacy was assessed using the 10-item General Self-Efficacy Scale [30]. Items are rated on a 4-point scale (1=not at all true and 4=exactly true), with total scores ranging from 10 to 40. Higher scores reflect stronger self-efficacy. In this study, the Cronbach α was .727.

### Experimental Procedures

The study was conducted in a psychobehavioral laboratory converted from a hospital treatment room, thereby reducing patient movement and ensuring safety. Sessions were scheduled for 4 PM on the day preceding surgery, a time selected to minimize potential disturbances. The experimental environment was controlled, with indoor lighting maintained at a constant level by adjusting room lights and drawing curtains to block external light. A dual-screen setup was employed to reduce participants’ awareness of being monitored. Eye movement data were collected using a Tobii Pro Fusion portable eye tracker. The experimental protocol was programmed using the Tobii Pro Lab software (version 24.21.435; Tobii AB, Stockholm, Sweden), and the stimuli were presented on a 23.8-inch Xiaomi Redmi monitor (resolution: 1920×1080 pixels), which could be vertically adjusted to accommodate participants’ seated height.

Prior to participation, informed consent procedures were conducted in a quiet setting, with research staff reading all study information aloud to participants to ensure comprehension. Adequate time was provided for questions, and comprehension was confirmed through teach-back techniques before written consent was obtained. To monitor fatigue during the experiment, trained staff members observed participants continuously, pausing the session if any signs of tiredness, distraction, or distress were detected. Participants were reminded of their right to withdraw at any time without consequence, and several short breaks were offered between tasks if needed.

Upon commencement, participants were seated 65 cm from the monitor. A 5-point calibration procedure was performed for each participant. Following successful calibration, participants were instructed to maintain head stability throughout the experiment. Each participant then completed 6 learning tasks corresponding to 6 different educational modules. The order of modules and their assigned modalities followed the 6×6 Latin square design, which ensured that across the sample, all modalities appeared equally in each serial position while preventing any single participant from encountering repeated content in different formats.

**Figure 2.**
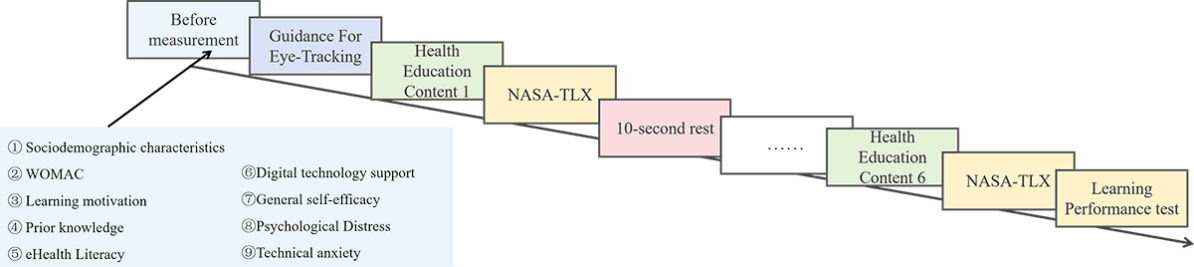
Experimental procedure of the 2 (high vs low learning motivation)×3 (text, text-graphic, video) factorial design study, conducted in a psychobehavioral laboratory with older patients scheduled for knee arthroplasty. NASA-TLX: National Aeronautics and Space Administration Task Load Index; WOMAC: Western Ontario and McMaster Universities Osteoarthritis Index.

During each learning task, the eye tracker recorded participants’ eye movement metrics. Upon completion of a module, participants clicked the mouse to advance to a blank screen and completed the NASA-TLX questionnaire to evaluate the cognitive load associated with that specific task. A subsequent mouse click initiated the next module. Upon completion of all 6 modules, participants undertook a post-learning test consisting of 30 single-choice questions according to the 6 learning modules (5 items per module). The sequence of the experimental procedure is illustrated in Figure 2.

### Ethical Considerations

This study was reviewed and approved by the Institutional Review Board of the Committee on the Ethics of Medical Research, Shanghai Guanghua Hospital affiliated to Shanghai University of Traditional Chinese Medicine (Approval No. 2023-K-45). All study procedures complied with the Declaration of Helsinki and relevant national biomedical ethics guidelines. Prior to participation, all individuals received detailed information regarding the study’s objectives, procedures, and potential risks and benefits. Written informed consent was obtained from all participants. They were informed of their right to decline participation or withdraw from the study at any point without penalty. To protect participants’ privacy, all collected data were anonymized at the point of entry, with identifying information removed. Data were stored on password-protected servers accessible only to the research team. Survey responses and eye-tracking records were coded with unique identifiers, ensuring that no individual participant could be traced from the dataset. Participants did not receive monetary compensation. To acknowledge their contribution, all participants were provided with a small educational gift (valued at less than 50 CNY [about 7 US $]) after completing the experiment. No identifying personal images or videos of participants are included in this study or its supplementary materials.

### Data Processing and Analysis

#### Eye Movement Data Processing

Eye movement data were collected using a Tobii Pro Fusion portable eye tracker. Following data acquisition, area of interests (AOIs) was defined within the Tobii Pro Lab software (File S5 in Multimedia Appendix 1 for details). Based on the distinct DHE content modules, patient eye movement data were analyzed across 6 predefined AOIs: anatomy of the knee joint, knee arthroplasty, implant selection, surgical procedure, rehabilitation exercises, and postoperative outcomes. For video-based presentations, AOIs included the main visual area, index, and subtitles. As the key eye movement, metrics average fixation duration, number of fixation points, and duration before the first fixation were extracted from the software and compiled into Excel files for subsequent statistical analysis.

#### Statistical Analysis

Data were analyzed using IBM SPSS Statistics (version 27.0; IBM Corp., Armonk, NY, USA). Descriptive statistics were generated for all variables. Continuous data were summarized using means and SD or medians and interquartile ranges, as appropriate. Categorical data were described using frequencies and percentages. To analyze the effects of independent variables on the dependent variables, analysis of covariance was performed within the general linear model framework to examine interaction effects. Statistically significant outcomes were further explored using Bonferroni-adjusted post hoc tests to identify specific differences among group means. Additionally, a 2-way ANOVA adjusted by Bonferroni was carried out using GraphPad Prism (version 10.1.2; GraphPad Software, Boston, MA, USA) to evaluate the impact of different levels of the independent variables on the dependent variable. Covariates for the analysis of covariance analyses were selected based on baseline differences. Specifically, any sociodemographic or psychosocial variables (including knee function, prior knowledge, eHealth literacy, psychological distress, technophobia, digital technology support, and self-efficacy) that showed a statistically significant difference (*P*<.05) between groups were included. The relationship between cognitive load and learning performance was examined using Pearson correlation analysis, performed in the R software (version 4.4.2; R Foundation for Statistical Computing, Vienna, Austria). Statistical significance was set at *P*<.05.

## Results

### Participant Characteristics and Descriptive Statistics

**Figure 3.**
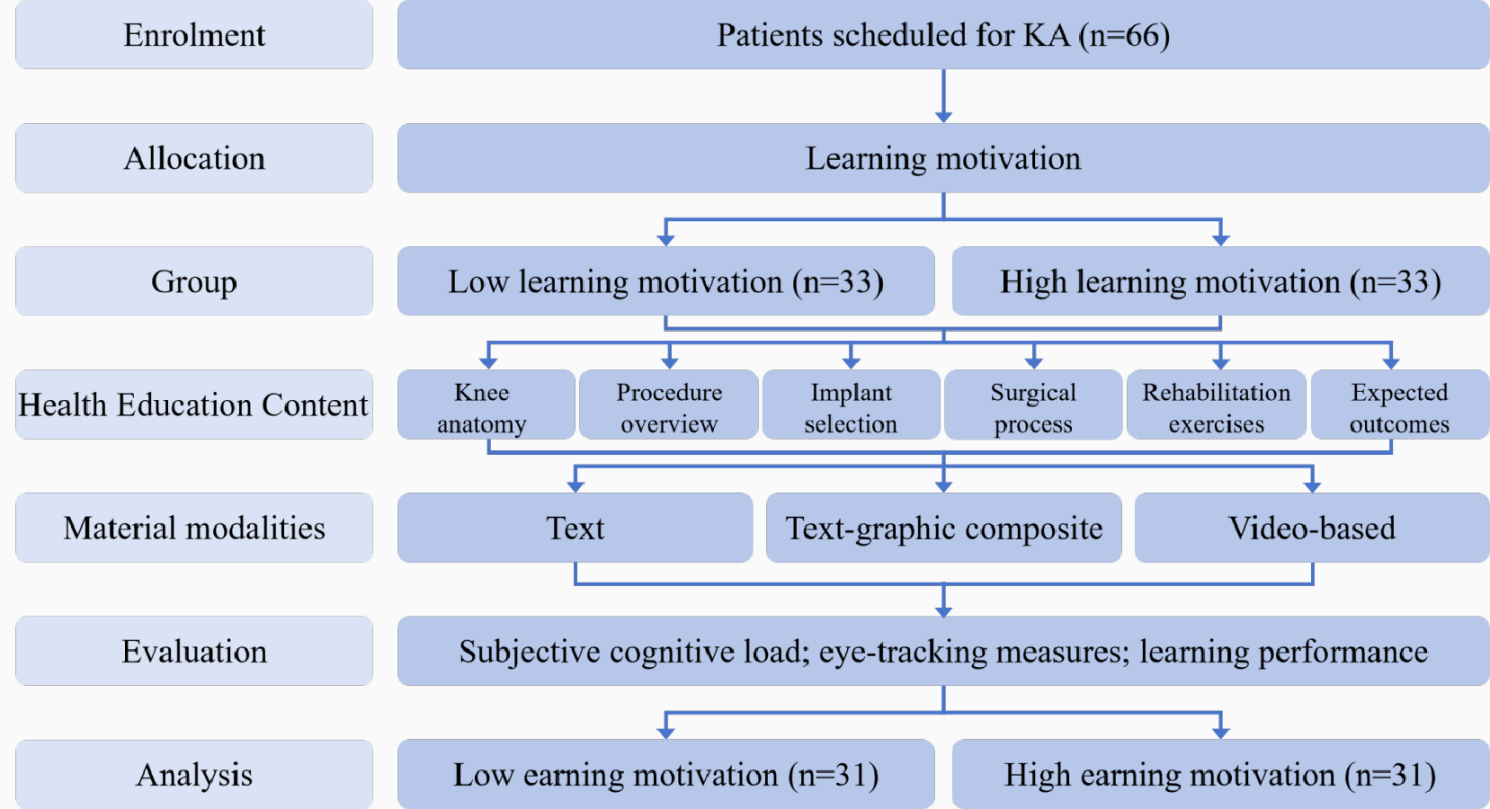
Flowchart of participant recruitment, screening, and inclusion in the study evaluating digital health education modalities among older patients with knee arthroplasty (KA) undergoing preoperative digital health education (conducted in Shanghai, China between July and December 2024).

A total of 66 participants were recruited between July 1, 2024, and December 25, 2024. At least 4 were excluded due to an effective eye-tracking data collection rate below 60%. The final analytical sample comprised 62 participants, with 31 in the low learning motivation group and 31 in the high learning motivation group. The study flow is illustrated in Figure 3.

At baseline, the mean learning motivation scores were 22.19 (SD 1.60) in the low-motivation group and 55.26 (SD 2.87) in the high-motivation group. Descriptive statistics for demographic characteristics and covariates related to cognitive load are presented in Table 1.

**Table 1.** Descriptive statistics of demographic characteristics and covariates among older patients with knee arthroplasty (KA) undergoing preoperative digital health education, stratified by learning motivation level (low vs high) (N=61).

Characteristics	Low (n=31)	High (n=31)	*t* test/*χ*^2^ (*df*)	*P* value
Total, n (%)	31 (100)	31 (100)		
Gender, n (%)				
Male	8 (25.8)	7 (22.6)	0.088 (1)	.77
Female	23 (74.2)	24 (77.4)		
Age (years), n (%)				
<70	10 (32.3)	17 (54.8)	3.215 (1)	.07
≥70	21 (67.7)	14 (45.2)		
Place of residence, n (%)				
Town	8 (25.8)	6 (19.4)	3.609 (1)	.17
Rural	9 (29.0)	4 (12.9)		
City	14 (45.2)	21 (67.7)		
Marital status, n (%)				
Married	27 (87.1)	28 (90.3)	−0.051^a^ (1)	.69
Other	4 (12.9)	3 (9.7)		
Degree of education, n (%)				
Primary school	18 (58.1)	17 (54.8)	0.066 (2)	.79
Middle school and above	13 (41.9)	14 (45.2)		
Employment status, n (%)				
Retirement	29 (93.5)	30 (96.8)	−0.075^a^ (1)	.55
Other	2 (6.5)	1 (3.2)		
Profession, n (%)				
Company employees	7 (22.6)	10 (32.3)	1.369 (2)	.50
Self-employed businessperson	12 (38.7)	13 (41.9)		
Other	12 (38.7)	8 (25.8)		
Average monthly income per capita of a household, n (%)				
≤3000 CNY^b^	13 (41.9)	6 (19.4)	0.251^a^ (2)	.14
3001‐5000 CNY	16 (51.6)	21 (67.7)		
≥5000 CNY	2 (6.5)	4 (12.9)		
Living alone, n (%)				
Yes	4 (12.9)	1 (3.2)	0.178^a^ (1)	.16
No	27 (87.1)	30 (96.8)		
Number of children, n (%)				
≤1	15 (48.4)	25 (80.7)	7.045 (1)	.09
>1	16 (51.6)	6 (19.3)		
Type of care, n (%)				
Family care	14 (45.2)	13 (41.9)	0.066 (1)	.80
Care by a caregiver	17 (54.8)	18 (58.1)		
BMI, n (%)				
<24	7 (22.6)	8 (25.8)	0.110 (2)	.95
≥24, <28	12 (38.7)	11 (35.5)		
≥28	12 (38.7)	12 (38.7)		
Principal diagnosis, n (%)				
Knee osteoarthritis	30 (96.8)	28 (90.3)	0.131^a^ (1)	.30
Rheumatoid arthritis	1 (3.2)	3 (9.7)		
Disease duration, n (%)				
<5	14 (45.2)	13 (41.9)	0.128 (2)	.94
5‐10	12 (38.7)	12 (38.7)		
>10	5 (16.1)	6 (19.4)		
Whether the first time for knee arthroplasty, n (%)				
Yes	25 (80.7)	23 (74.2)	0.369 (1)	.54
No	6 (19.3)	8 (25.8)		
The area to be operated on, n (%)				
Left	12 (38.7)	15 (48.4)	0.590 (1)	.44
Right	19 (61.3)	16 (51.6)		
Type of proposed surgery, n (%)				
Unicompartmental knee arthroplasty	15 (48.4)	16 (51.6)	0.065 (1)	.80
Total knee arthroplasty	16 (51.6)	15 (48.4)		
Comorbid diseases, n (%)				
Yes	23 (74.2)	24 (77.4)	0.088 (1)	.77
No	8 (25.8)	7 (22.6)		
Western Ontario and McMaster Universities Osteoarthritis Index (WOMAC), mean (SD)	113.06 (16.66)	118.84 (18.61)	−1.287 (60)	.20
Learning motivation, mean (SD)	22.19 (1.60)	55.26 (2.87)	−55.952 (60)	<.001
Prior knowledge, mean (SD)	7.68 (2.81)	8.29 (2.65)	−0.884 (60)	.38
eHealth literacy, mean (SD)	24.45 (6.95)	27.52 (6.15)	−1.838 (60)	.07
Self-efficacy, mean (SD)	25.26 (5.83)	27.32 (5.61)	−1.420 (60)	.16
Psychological distress, mean (SD)	15.03 (2.27)	14.48 (2.35)	0.934 (60)	.35
Technophobia, mean (SD)	34.81 (11.16)	32.00 (9.74)	1.055 (60)	.30

a Fisher’s exact probability method.

b All income data were reported in Chinese Renminbi (RMB). For reference, the average exchange rate at the time of the study was 1 US $ ≈ 7.19 CNY.

Across all participants, the mean subjective cognitive load (NASA-TLX) was 31.23 (SD 6.36). The average fixation duration was 247.64 (SD 55.04) ms, the number of fixation points was 106.69 (SD 34.30), and the duration before the first fixation was 274.47 (SD  74.10) ms. The average learning performance score was 2.73 (SD 1.12) out of a maximum of 5. A detailed breakdown of subjective and objective cognitive load metrics, as well as learning performance across different presentation modalities and educational modules, is provided in Table S1 in Multimedia Appendix 1. Relationships between covariates and dependent variables are provided in Table S2 in Multimedia Appendix 1.

### Effects of Learning Motivation and Presentation Modality

A general linear model was used to assess the main and interaction effects of learning motivation (low vs high) and presentation modality (text, text-graphic composite, and video-based) on subjective cognitive load (NASA-TLX), objective cognitive load (average fixation duration, number of fixation points, and duration before first fixation), and learning performance (Table 2).

**Table 2.** Main and interaction effects of learning motivation and educational material modalities on cognitive load and learning performance in older KA patients undergoing preoperative digital health education (N=372).

Dependent variable	Independent variable	SS^a^	MS^b^	*F* test (*df*)	*P* value	*η*²_p_
National Aeronautics and Space Administration Task Load Index (NASA-TLX)	Covariates (number of children)	110.173	110.173	3.072 (1)	.08	0.008
	Learning motivation	1349.172	1349.172	37.625^c^ (1)	<.001	0.093
	Presentation modalities	470.296	235.148	6.558^d^ (2)	.002	0.035
	Interactions	86.231	43.116	1.202 (2)	.30	0.007
	Error (within groups)	13,088.408	35.859			
Average fixation duration	Covariates (number of children)	456.735	456.735	0.163 (1)	.68	0.000
	Learning motivation	61,794.009	61,794.009	22.074^c^ (1)	<.001	0.057
	Presentation modalities	32,816.468	16,408.234	5.861^d^ (2)	.003	0.031
	Interactions	2989.263	1494.632	0.534 (2)	.58	0.003
	Error (within groups)	1,021,783.604	2799.407			
Number of fixation points	Covariates (number of children)	322.800	322.800	0.292 (1)	.59	0.001
	Learning motivation	4704.020	4704.020	4.259^e^ (1)	.04	0.012
	Presentation modalities	24,341.952	12,170.976	11.019^c^(2)	<.001	0.057
	Interactions	4344.726	2172.363	1.967	.14	0.011
	Error (within groups)	403,161.539	1104.552			
Duration before the first fixation	Covariates (number of children)	645.340	645.340	0.131 (1)	.72	0.000
	Learning motivation	131,729.141	131,729.141	26.737^c^ (1)	<.001	0.068
	Presentation modalities	54,117.919	27,058.960	5.492^d^ (2)	.004	0.029
	Interactions	42,409.468	21,204.734	4.304^e^ (2)	.01	0.023
	Error (Within Groups)	1,798,320.789	4926.906			
Learning performance	Covariates (number of children)	3.058	3.058	2.660 (1)	.10	0.007
	Learning motivation	39.085	39.085	34.000^c^ (1)	<.001	0.085
	Presentation modalities	4.215	2.108	1.833 (2)	.16	0.010
	Interactions	0.086	0.043	0.037 (2)	.96	0.000
	Error (within groups)	419.587	1.150			

a SS: sum of squares.

b MS: mean squares.

c *P*<.001.

d *P*<.01.

e *P*<.05.

Significant main effects were observed for both learning motivation and presentation modality on subjective cognitive load (*F*_(1, 365)_=37.625, *P*<.001, *η*²_p_=0.093; *F*_(1, 365)_=6.558, *P*=.002, *η*²_p_=0.035, respectively). Similarly, both variables exerted significant effects on objective cognitive load indicators: (1) average fixation duration: *F*_(1, 365)_=22.074, *η*²_p_=0.057 (motivation), *F*_(1, 365)_  = 5.861, *η*²_p_=0.031 (modality), both *P*<.001; (2) number of fixation points: *F*_(1, 365)_  = 4.259, *η*²_p_=0.012 (motivation), *P*=.04; *F* _(1, 365)_=11.019, *η*²_p_=0.057 (modality), *P*<.001.

**Figure 4.**
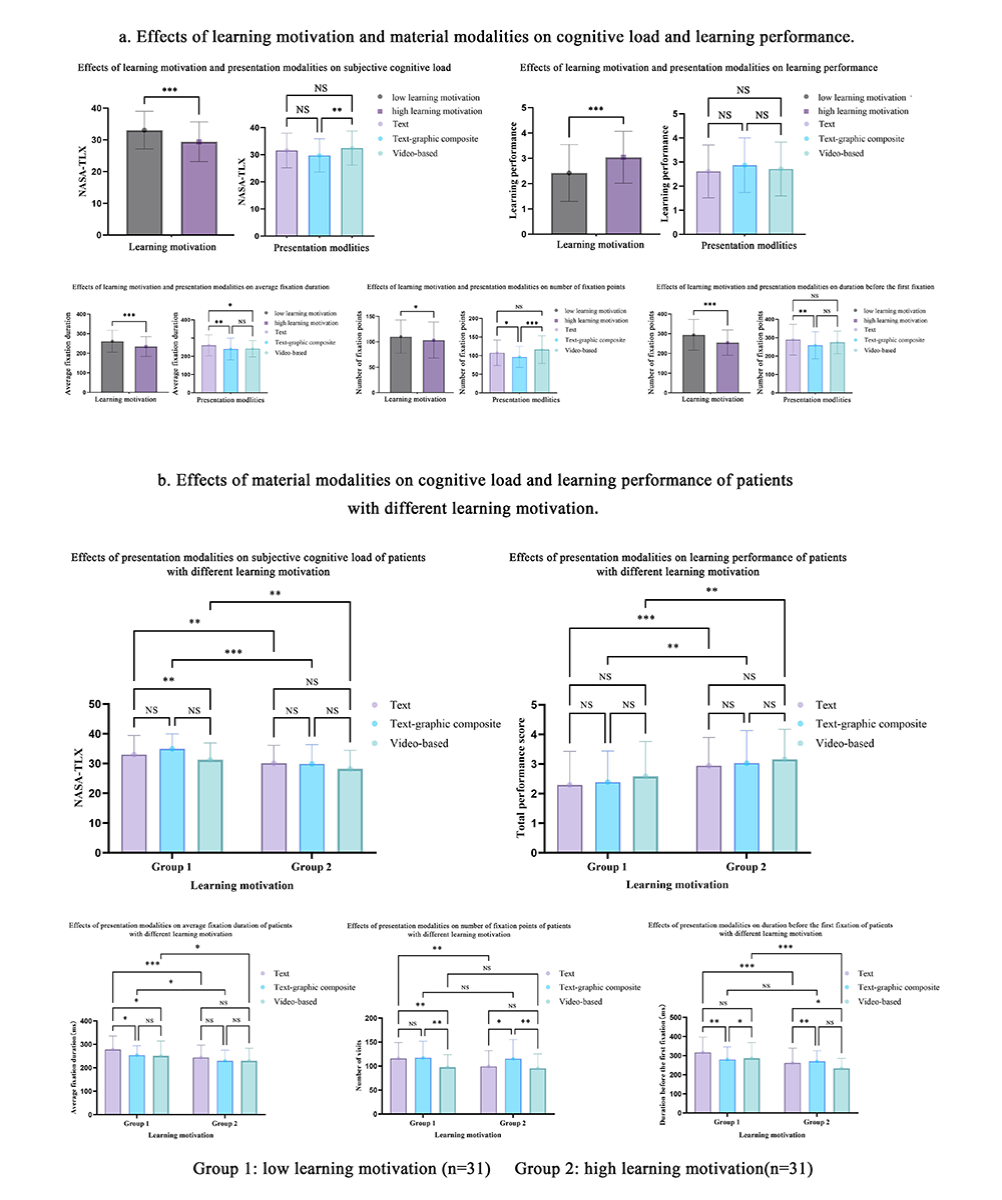
Differences in cognitive load and learning performance across learning motivation levels and presentation modalities among older patients with knee arthroplasty undergoing preoperative digital health education (N=372). Error bars represent standard errors; asterisks denote significance levels: *P<.05, **P<.01, ***P<.001; multiple comparison adjustment: Bonferroni method.

Post hoc comparisons revealed that patients with low learning motivation experienced significantly higher subjective cognitive load, longer fixation durations, and fewer fixation points than their high-motivation counterparts. Moreover, the effects of presentation modality on cognitive load were more pronounced in the low-motivation group (Figure 4). For detailed information on post hoc comparisons, refer to Tables 3 and 4 (Multimedia Appendix 1). Learning performance was significantly influenced by learning motivation (*F*_(1, 365)_=34.000, *P*<.001), while the main effect of presentation modality on learning performance did not reach statistical significance.

### Correlation Between Cognitive Load and Learning Performance

**Figure 5.**
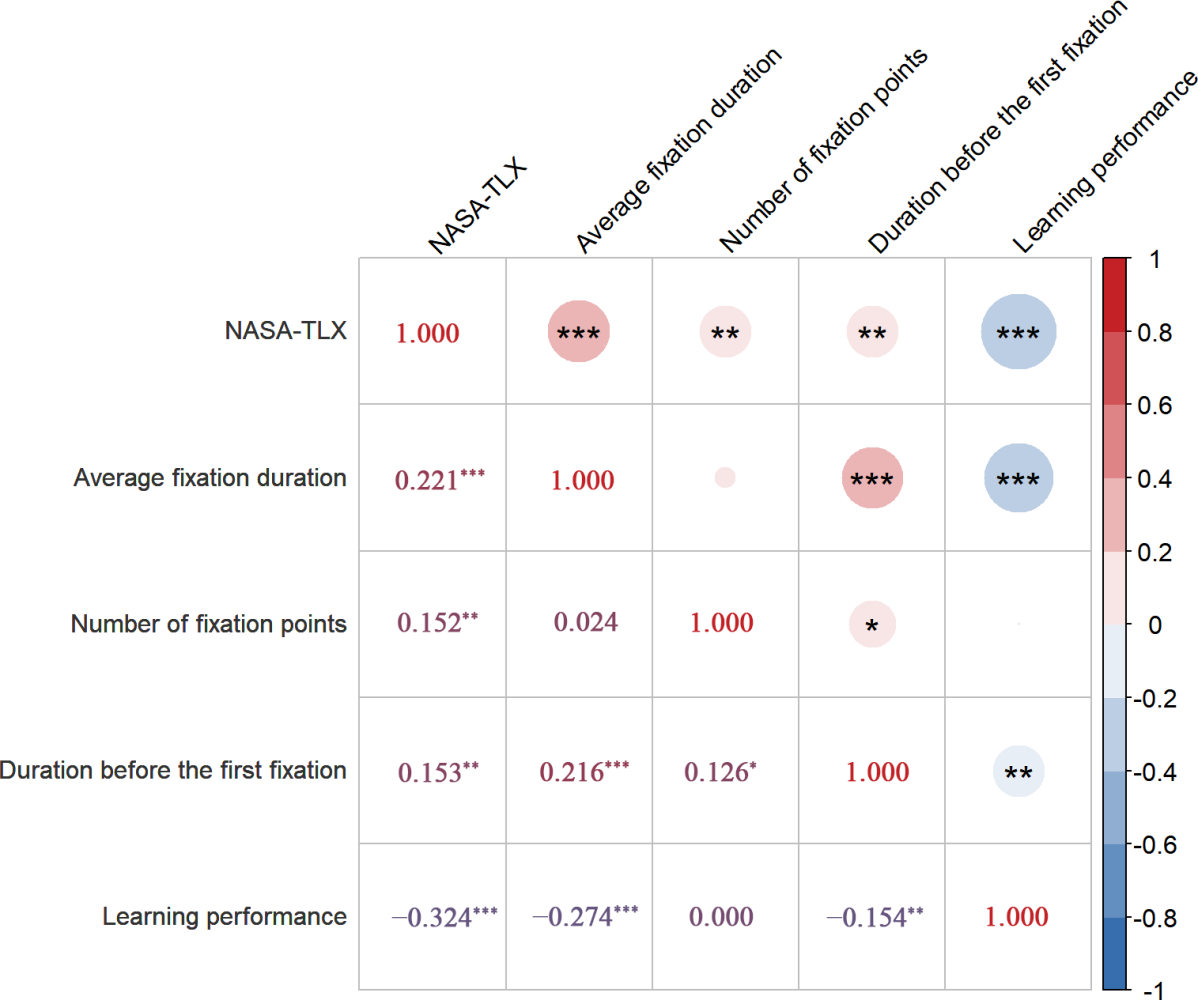
Pearson correlation analysis between cognitive load and learning performance in older patients with knee arthroplasty undergoing preoperative digital health education (N=372). Note: On the right side of 1.00 is the *P* value, and on the left side is the *r* value; *P<.05; **P<.01, ***P<.001.

Pearson correlation analyses were conducted to explore the relationships between cognitive load metrics and learning performance. The results, shown in Figure 5, indicate significant negative correlations between learning performance and: (1) subjective cognitive load (NASA-TLX): *r *= −0.32, *P*<.001; (2) average fixation duration: *r* = −0.27, *P*<.001; (3) time before first fixation: *r* = −0.15, *P*=.003. For specific details, refer to Table S5 (Multimedia Appendix 1).

## Discussion

### Key Findings

To our knowledge, this is the first study to investigate factors influencing learning outcomes in DHE for older adult patients undergoing KA using a psychobehavioral experimental paradigm. The results demonstrate that both presentation modality and learning motivation significantly affect cognitive load and learning performance. Rather than reflecting simple media differences, these findings can be understood through specific mechanisms within CLT. Text-graphic composite formats elicited higher fixation-based load but also supported effective learning, consistent with the segmentation effect, which emphasizes that chunking complex information fosters schema construction [11,31]. Video-based materials minimized extraneous load and facilitated comprehension, reflecting the modality effect, which shows that distributing information across auditory and visual channels enhances processing efficiency [32]. Notably, high-learning motivation served as a protective factor, supporting germane load by sustaining attention and schema-building. While the interaction between motivation and modality was explored, it did not reach significance across all outcomes; therefore, our discussion emphasizes main effects, with interaction patterns interpreted cautiously. These findings reinforce the importance of grounding digital education design in CLT principles rather than media comparisons, highlighting the need for cognitively aligned and personalized strategies.

This study provides empirical evidence that learning motivation significantly influences both cognitive load and learning outcomes in preoperative DHE for patients undergoing KA. Participants with high motivation demonstrated lower cognitive load and superior performance, supporting the role of motivation in facilitating germane load allocation within CLT [8,33]. Intrinsically motivated learners appear more capable of regulating attention, sustaining schema construction, and engaging in deeper learning strategies, even under cognitively demanding conditions. In the complex preoperative context, where rapid assimilation of procedural and rehabilitation knowledge is critical, high motivation enhances patients’ self-regulation and cognitive resilience. These findings align with prior evidence that motivational deficits impede engagement with digital health interventions in older adults [34], underscoring motivation as a protective factor in cognitively challenging learning environments.

Regarding presentation modality, the text-graphic composite format generated higher cognitive load yet still fostered effective knowledge retention, consistent with the segmentation effect, which enables learners to gradually integrate textual and visual elements [11]. Although elderly patients may experience greater visual effort due to age-related decline or suboptimal integration of multimodal input [35,36], the combined use of text and graphics supports dual-channel processing and promotes schema construction [10]. The increased load observed in this condition likely reflects a productive rather than detrimental challenge, aligning with recent perspectives in cognitive load research that emphasize the optimization, rather than mere reduction, of load in complex learning tasks [12]. Thus, the composite format may encourage deeper processing despite imposing higher cognitive demands, particularly when appropriately segmented and scaffolded.

In contrast, the video-based format was associated with the lowest cognitive load and the highest learning performance, consistent with the modality effect, which highlights the benefits of distributing information across auditory and visual channels [10]. Dynamic visual delivery reduced ambiguity, sustained attention, and facilitated procedural understanding, particularly for sequential demonstrations such as rehabilitation exercises [4]. However, this format also presents age-related challenges: for older or less-prepared learners, the transient information effect may arise when rapidly disappearing elements overload working memory unless appropriately segmented or learner-controlled [12]. To address this risk, patient education videos should incorporate features such as adjustable playback speed, captions, and clear sequencing to balance cognitive demands across diverse learner profiles. Properly designed, video-based materials can reduce extraneous load while supporting effective schema construction in digital health education.

Although exploratory analyses suggested possible patterns of interaction between motivation and presentation modality, these effects were not consistently significant across outcomes. Patients with high motivation demonstrated consistent outcomes across formats, likely reflecting adaptive metacognitive strategies such as selective attention and self-monitoring that buffered against extraneous load [37]. This aligns with the role of germane load, whereby motivation facilitates schema construction and deeper learning. In contrast, low-motivation learners were more sensitive to modality-related challenges, suggesting a greater risk of overload when materials were not optimally segmented or scaffolded. Given the nonsignificant interaction results, these interpretations should be viewed as tentative. Prior research cautions that high motivation does not invariably enhance learning; when content is excessively demanding or poorly aligned, motivation can amplify frustration and information overload, paradoxically increasing cognitive load [38]. These findings underscore the need for tailored instructional designs that integrate CLT principles, minimizing extraneous load through modality, managing intrinsic load through segmentation, and fostering germane load through motivation, to optimize digital health education.

Our results confirm a negative correlation between cognitive load and learning performance, with temporal sequencing allowing inference of a directional influence: cognitive load impacts learning outcomes. This reinforces the role of cognitive load as a mediating factor in learning, with higher load generally impairing outcomes. Although some literature suggests a curvilinear (inverted-U) relationship between cognitive load and learning [39], our results are consistent with the linear-negative pattern commonly observed in clinical education contexts. The absence of performance deterioration under higher cognitive load in the text-graphic condition may reflect the effectiveness of optimized material design or the fact that load levels did not exceed patients’ cognitive thresholds.

### Implications

#### Enhancing Patient Learning Motivation

This study highlights the importance of learning motivation as a determinant of cognitive efficiency and learning success in preoperative DHE. Clinicians should adopt strategies to boost patient motivation by emphasizing the personal relevance and clinical benefits of the educational content. Tailoring materials to individual interests, incorporating interactive elements, and leveraging behavior change theories may further enhance motivation, especially in older adults [34]. Additionally, family-based or intergenerational learning models may provide emotional support, reduce digital anxiety, and foster sustained engagement. Importantly, while motivation is generally beneficial, its effects are not universally linear. When material complexity exceeds a learner’s capacity, heightened motivation may paradoxically intensify cognitive load and reduce performance [39]. Therefore, motivation enhancement strategies must be tailored to individual readiness and cognitive resources.

#### Optimizing the “Learner-Presentation Format” Fit

Effective health education requires careful alignment between learner characteristics and content presentation. This study underscores the need to develop an adaptive “learner-presentation format” matching framework, incorporating variables such as age, health literacy, digital fluency, and disease complexity [6]. Such a model would enable the selection of presentation formats that minimize extraneous load and maximize learning efficiency. For instance, patients with strong digital skills and health literacy may benefit from interactive video content, whereas those with higher technophobia or lower literacy may require simplified text-graphic formats with progressive scaffolding [40]. While this principle is universal, our study was conducted in Shanghai, where digital penetration through mobile platforms (eg, WeChat-based health services) is high and family involvement in medical decisions is common. In contrast, Western health care contexts may rely more on institutional e-learning systems and emphasize individual decision-making. Therefore, adapting the “learner-presentation format” fit to cultural, technological, and institutional conditions will be essential for broader applicability.

#### Defining and Dynamically Managing Cognitive Load Thresholds

Our results confirm that both presentation modality and learning motivation significantly shape cognitive load and learning outcomes, underscoring the importance of identifying and managing cognitive load thresholds in DHE. Recent advances in eye-tracking, electroencephalography, and multimodal analytics suggest that cognitive load can be monitored in near real time, enabling adaptive interventions that segment complex material, slow pacing, or provide supportive cues when overload is detected [12,36]. While physiological and behavioral measures show promise, feasibility in clinical education requires minimally intrusive, scalable tools [41]. We propose that future adaptive systems, guided by CLT principles, dynamically balance modality, segmentation, and transient information to keep learners within optimal load ranges, thereby fostering schema construction without inducing overload [32].

### Limitations

Several limitations must be acknowledged. First, participant recruitment was confined to a single orthopedic specialty hospital in eastern China. While the hospital ranks among the region’s top institutions in surgical volume, the single-center design limits generalizability. Future studies should incorporate multisite recruitment to enhance representativeness. Second, although the readability of educational materials was strictly controlled to accommodate the cognitive vulnerabilities and preoperative stress of older adult patients, variation in individual reading skills could still have influenced comprehension and learning performance. This unmeasured factor should be addressed in future studies by incorporating standardized assessments of literacy or tailoring interventions to different reading proficiency levels. Third, while this study investigated 3 common presentation formats, it did not explore intraformat variation (eg, image complexity or text density) that may influence cognitive load. Further research should draw on multimedia learning principles to examine the nuanced design features affecting learner outcomes. Fourth, only short-term learning performance was assessed, and the reliability of module-level test scores was constrained by the small number of items per module and the heterogeneous nature of content. These design choices, made to reduce cognitive burden on elderly surgical patients, resulted in modest KR-20 values despite satisfactory content validity, item difficulty, and discrimination indices. Future studies should employ more comprehensive assessment tools with adequate item numbers to improve reliability while balancing participant burden. Fifth, cognitive load was measured using the Chinese version of the NASA-TLX. Although this instrument enabled reliable assessment of overall cognitive load, it does not differentiate between intrinsic, extraneous, and germane load. More recent instruments provide this granularity but have not yet been fully adapted or validated for Chinese clinical populations. Future research should incorporate such multidimensional measures, once culturally adapted, to better disentangle sources of cognitive load in digital health education. Finally, despite efforts to reduce burden through optimized materials and unobtrusive eye-tracking, the overall length and complexity of the protocol, including multiple questionnaires, may still have induced some survey fatigue. This limitation is inherent to conducting real-world research with older adult surgical patients, a vulnerable population, but it also underscores the unique theoretical and practical contributions of implementing digital health education in authentic clinical contexts.

### Conclusions

This study employed a psychobehavioral experimental design to investigate the interactive effects of learning motivation and presentation modality on cognitive load and learning performance in preoperative DHE for knee arthroplasty patients. The results demonstrate that both learning motivation and presentation format significantly influence cognitive processing and educational outcomes. Patients with high learning motivation exhibited lower cognitive load and better learning outcomes. Among the presentation formats, video-based materials led to the lowest cognitive load and the highest performance. A consistent negative correlation was observed between cognitive load and learning performance, reinforcing the central role of cognitive load management in DHE design. Future research should aim to stimulate patient learning motivation, refine learner-format matching models, and develop intelligent systems capable of dynamically regulating cognitive load to optimize educational effectiveness across diverse patient populations.

## Supplementary material

10.2196/79430 Multimedia Appendix 1 Supplementary material, including all supplementary figures, tables, and files.

## Abbreviations

AOI: area of interest
CLT: Cognitive Load Theory
DHE: digital health education
KA: knee arthroplasty
KR-20: Kuder-Richardson 20
NASA-TLX: National Aeronautics and Space Administration Task Load Index
