# Supplementary material for: Impact of Learning Motivation and Presentation Modalities on Cognitive Load and Learning Performance in Preoperative Digital Health Education for Older Patients With Knee Arthroplasty: Psychobehavioral Experimental Study
Source: J Med Internet Res. 2025 Oct 24;27:e79430. doi: 10.2196/79430 (PMC12551793; doi:10.2196/79430)

Supplement Figures

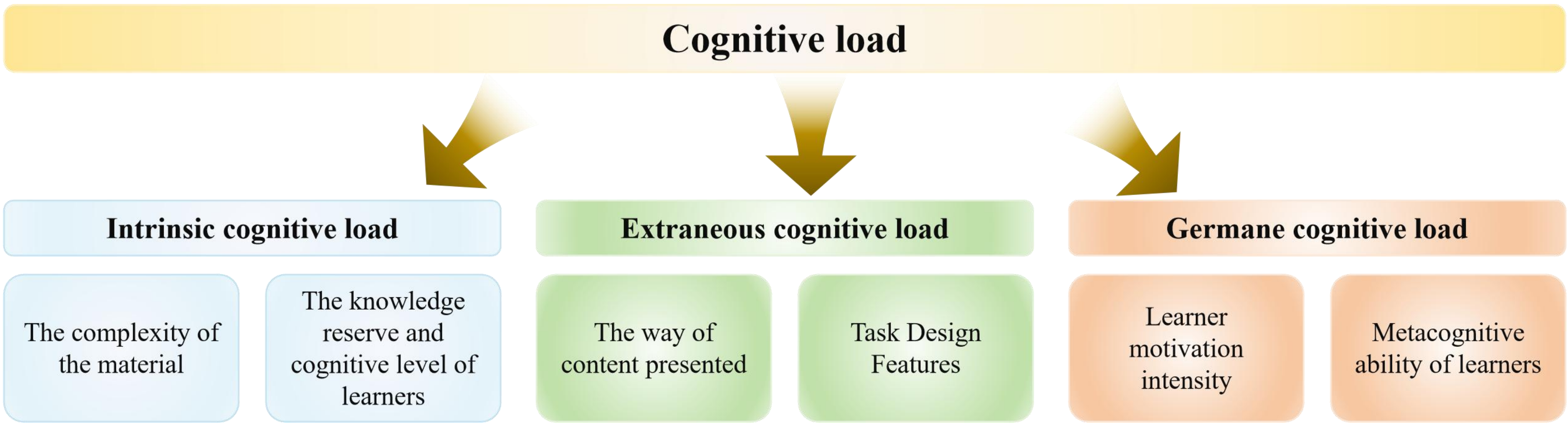

Supplement Figure 1 Cognitive load theory and its application in patient health education

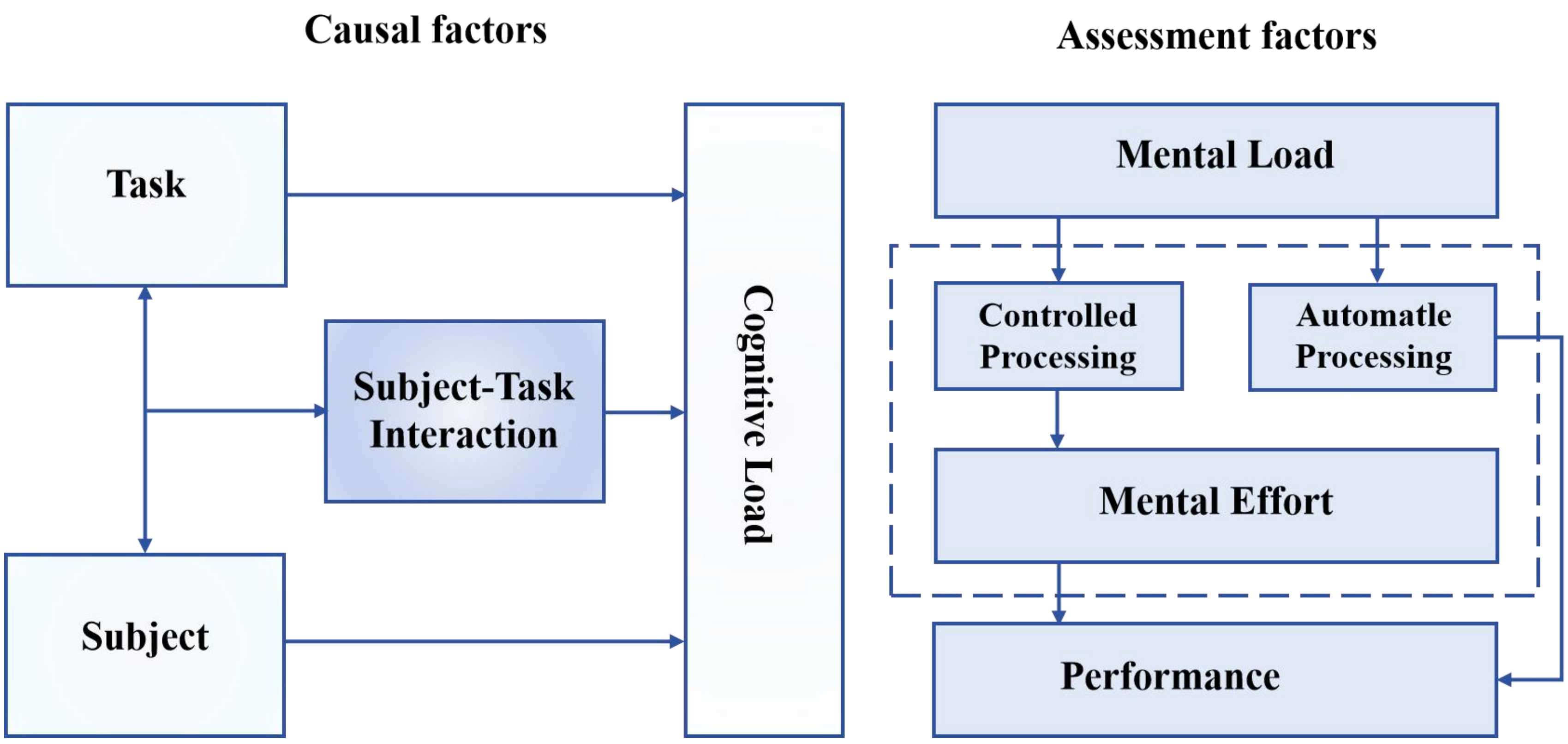

Supplement Figure 2 Two-dimensional structural model of cognitive load

Supplement Table

Supplement Table 1 Cognitive load and learning performance scores across different presentation modalities and health education modules in older knee arthroplasty patients (N=372).

Variable name			Cognitive load, mean (SD)				Learning performance, mean (SD)
Health education contents	Learning motivation	Material modalities	NASA-TLX,	Average fixation duration	Number of fixation points	Duration before the first fixation	
Knee anatomy	Low(n=31)	Text	34.44 (4.80)	324.33 (39.42)	127.67 (23.61)	348.56 (64.83)	1.89 (0.93)
		Text-graphic composite	34.00 (5.93)	259.10 (43.56)	119.00 (37.87)	264.40 (61.59)	3.00 (1.05)
		Video-based	31.33 (6.36)	242.17 (69.37)	96.83 (39.42)	304.58 (91.98)	2.50 (1.45)
		Total	33.10 (5.79)	271.48 (63.10)	112.94 (36.47)	304.39 (80.50)	2.48 (1.23)
	High(n=31)	Text	28.50 (6.55)	262.40 (55.23)	99.70 (33.15)	247.90 (63.00)	2.70 (0.82)
		Text-graphic composite	27.92 (6.05)	236.69 (45.22)	111.23 (40.78)	285.77 (51.94)	3.62 (1.19)
		Video-based	29.63 (6.19)	247.38 (28.87)	106.38 (39.22)	221.13 (46.34)	2.87 (0.64)
		Total	28.55 (6.08)	247.74 (45.30)	106.26 (37.13)	256.87 (59.17)	3.13 (1.02)
	Total(n=62)	Text	31.32 (6.40)	291.74 (56.79)	112.95 (31.67)	295.58 (80.74)	2.32 (0.95)
		Text-graphic composite	30.57 (6.62)	246.43 (44.95)	114.61 (38.85)	276.48 (56.05)	3.35 (1.15)
		Video-based	30.65 (6.18)	244.25 (55.68)	100.65 (38.59)	271.20 (86.31)	2.65 (1.18)
		Total	30.82 (6.32)	259.61 (55.77)	109.60 (36.65)	280.63 (74.04)	2.81 (1.17)
Procedure overview	Low(n=31)	Text	34.15 (8.18)	278.08 (56.52)	139.54 (33.87)	365.00 (67.93)	2.46 (1.45)
		Text-graphic composite	38.10 (6.82)	279.10 (56.89)	105.80 (41.78)	290.80 (60.93)	2.10 (1.10)
		Video-based	29.25 (6.73)	234.13 (42.48)	89.88 (16.38)	318.75 (93.83)	2.63 (1.19)
		Total	34.16 (7.93)	267.06 (55.32)	115.84 (38.75)	329.13 (78.04)	2.39 (1.26)
	High(n=31)	Text	31.09 (7.41)	256.00 (56.86)	107.64 (42.87)	295.45 (72.24)	3.00 (1.34)
		Text-graphic composite	31.57 (6.45)	238.14 (52.22)	114.57 (54.21)	304.71 (76.79)	2.86 (1.07)
		Video-based	26.54 (5.84)	215.69 (58.74)	88.92 (22.37)	215.62 (35.63)	3.15 (1.14)
		Total	29.29 (6.78)	235.06 (57.69)	101.35 (39.02)	264.06 (72.05)	3.03 (1.17)
	Total(n=62)	Text	32.75 (7.83)	267.96 (56.56)	124.92 (40.76)	333.13 (77.00)	2.71 (1.40)
		Text-graphic composite	35.41 (7.26)	262.24 (57.22)	109.41 (45.87)	296.53 (65.95)	2.41 (1.12)
		Video-based	27.57 (6.18)	222.71 (52.78)	89.29 (19.86)	254.90 (80.48)	2.95 (1.16)
		Total	31.73 (7.72)	251.06 (58.33)	108.60 (39.25)	296.60 (81.38)	2.71 (1.25)
Implant selection	Low(n=31)	Text	28.44 (6.75)	239.78 (61.57)	96.11 (48.50)	248.67 (60.12)	2.44 (1.51)
		Text-graphic composite	33.90 (4.43)	261.20 (41.71)	110.30 (24.45)	297.90 (68.39)	2.40 (1.07)
		Video-based	30.50 (4.06)	313.58 (60.34)	92.08 (33.27)	311.83 (83.75)	2.17 (1.27)
		Total	31.00 (5.38)	275.26 (62.44)	99.13 (35.73)	289.00 (75.24)	2.32 (1.25)
	High(n=31)	Text	28.27 (5.29)	210.00 (41.94)	89.36 (25.91)	210.82 (100.56)	3.18 (0.87)
		Text-graphic composite	31.83 (7.22)	233.50 (55.65)	115.67 (33.54)	281.17 (45.71)	2.67 (1.07)
		Video-based	30.00 (4.81)	257.38 (54.02)	111.13 (51.62)	284.00 (63.38)	2.87 (1.36)
		Total	30.10 (6.02)	231.32 (52.47)	105.16 (37.46)	256.94 (79.27)	2.90 (1.08)
	Total(n=62)	Text	28.35 (5.82)	223.40 (52.47)	92.40 (36.82)	227.85 (84.95)	2.85 (1.23)
		Text-graphic composite	32.77 (6.07)	246.09 (50.67)	113.23 (29.20)	288.77 (56.32)	2.55 (1.06)
		Video-based	30.30 (4.26)	291.10 (63.10)	99.70 (41.40)	300.70 (75.74)	2.45 (1.32)
		Total	30.55 (5.68)	253.29 (61.33)	102.15 (36.43)	272.97 (78.33)	2.61 (1.19)
Surgical process	Low(n=31)	Text	34.91 (7.31)	275.55 (54.05)	103.00 (25.79)	305.45 (67.01)	2.55 (0.93)
		Text-graphic composite	34.36 (3.07)	241.00 (29.46)	128.27 (33.16)	254.27 (94.06)	2.27 (1.01)
		Video-based	29.67 (5.34)	207.78 (37.45)	105.22 (19.52)	206.56 (43.50)	3.56 (0.88)
		Total	33.19 (5.82)	243.61 (48.98)	112.61 (28.81)	258.58 (81.08)	2.74 (1.06)
	High(n=31)	Text	32.56 (6.67)	255.00 (43.48)	111.00 (40.19)	309.22 (57.50)	2.56 (0.73)
		Text-graphic composite	29.00 (8.37)	229.89 (30.81)	93.67 (27.09)	278.78 (71.24)	3.00 (1.41)
		Video-based	26.85 (7.97)	226.69 (52.11)	88.69 (25.83)	235.92 (55.92)	3.23 (0.73)
		Total	29.13 (7.86)	235.84 (44.72)	96.61 (31.39)	269.65 (66.88)	2.97 (0.98)
	Total(n=62)	Text	33.85 (6.95)	266.30 (49.43)	106.60 (32.36)	307.15 (61.31)	2.55 (0.83)
		Text-graphic composite	31.95 (6.48)	236.00 (29.81)	112.70 (34.64)	265.30 (83.36)	2.60 (1.23)
		Video-based	28.00 (7.01)	218.95 (46.65)	95.45 (24.41)	223.91 (52.21)	3.36 (0.79)
		Total	31.16 (7.16)	239.73 (46.68)	104.61 (30.95)	264.11 (73.92)	2.85 (1.02)

Continued Supplement Table 1 Cognitive load and learning performance scores across different presentation modalities and health education modules in older knee arthroplasty patients (N=372).

Variable name			Cognitive load, mean (SD)				Learning performance, mean (SD)
Health education contents	Learning motivation	Material modalities	NASA-TLX,	Average fixation duration	Number of fixation points	Duration before the first fixation	
Rehabilitation exercises	Low(n=31)	Text	33.57 (3.76)	294.50 (54.24)	112.43 (26.35)	317.50 (87.58)	1.86 (0.86)
		Text-graphic composite	33.55 (4.80)	244.18 (30.47)	119.00 (38.18)	281.36 (45.33)	2.09 (1.04)
		Video-based	29.50 (4.76)	240.00 (59.54)	89.67 (18.37)	240.50 (38.43)	2.83 (0.41)
		Total	32.77 (4.50)	266.10 (53.52)	110.35 (30.95)	289.77 (71.59)	2.13 (0.92)
	High(n=31)	Text	31.00 (4.18)	263.92 (46.23)	97.67 (30.83)	255.17 (71.99)	2.67 (0.78)
		Text-graphic composite	33.25 (3.99)	229.25 (32.19)	118.50 (27.71)	259.50 (47.60)	2.50 (0.53)
		Video-based	27.91 (5.41)	202.64 (39.68)	92.00 (17.57)	199.00 (21.52)	3.73 (1.10)
		Total	30.48 (4.95)	233.23 (47.69)	101.03 (27.32)	236.35 (58.13)	3.00 (1.00)
	Total(n=62)	Text	32.38 (4.09)	280.38 (52.08)	105.62 (28.91)	288.73 (85.28)	2.23 (0.91)
		Text-graphic composite	33.42 (4.36)	237.89 (31.25)	118.79 (33.29)	272.16 (46.33)	2.26 (0.87)
		Video-based	28.47 (5.10)	215.82 (49.31)	91.18 (17.31)	213.65 (34.19)	3.41 (1.00)
		Total	31.63 (4.83)	249.66 (52.93)	105.69 (29.33)	263.06 (70.05)	2.56 (1.05)
Expected outcomes	Low(n=31)	Text	30.33 (5.43)	232.50 (29.13)	107.00 (9.88)	284.67 (91.05)	2.83 (0.75)
		Text-graphic composite	35.90 (4.12)	241.00 (31.38)	118.70 (34.42)	288.60 (62.51)	2.50 (0.97)
		Video-based	34.47 (5.85)	246.60 (56.60)	105.20 (16.35)	299.33 (69.22)	2.27 (1.03)
		Total	34.13 (5.48)	242.06 (44.28)	109.90 (23.13)	293.03 (69.50)	2.45 (0.96)
	High(n=31)	Text	29.11 (6.23)	210.89 (51.40)	90.67 (23.76)	258.00 (62.08)	3.56 (0.88)
		Text-graphic composite	27.85 (5.32)	214.00 (52.24)	132.31 (48.53)	227.15 (17.83)	3.23 (0.93)
		Video-based	30.11 (6.49)	247.67 (67.98)	93.78 (24.80)	260.67 (50.87)	2.89 (0.93)
		Total	28.87 (5.82)	222.87 (57.36)	109.03 (40.77)	245.84 (45.90)	3.23 (0.92)
	Total(n=62)	Text	29.60 (5.75)	219.53 (43.96)	97.20 (20.64)	268.67 (73.12)	3.27 (0.88)
		Text-graphic composite	31.35 (6.25)	225.74 (45.59)	126.39 (42.63)	253.87 (52.36)	2.91 (1.00)
		Video-based	32.83 (6.34)	247.00 (59.65)	100.92 (20.21)	284.83 (64.67)	2.50 (1.02)
		Total	31.50 (6.20)	232.47 (51.73)	109.47 (32.88)	269.44 (63.07)	2.84 (1.01)
Total	Low(n=186)	Text	33.00 (6.44)	278.08 (57.48)	115.76 (33.32)	316.66 (80.23)	2.29 (1.14)
		Text-graphic composite	34.94 (5.03)	253.89 (40.56)	117.06 (34.70)	279.18 (66.36)	2.39 (1.05)
		Video-based	31.24 (5.73)	250.82 (63.69)	97.56 (26.32)	286.11 (82.39)	2.58 (1.18)
		Total	33.06 (5.92)	260.93 (55.85)	110.13 (32.73)	293.98 (77.97)	2.42 (1.12)
	High(n=186)	Text	30.08 (6.03)	243.71 (52.87)	99.21 (33.06)	261.53 (77.59)	2.94 (0.96)
		Text-graphic composite	29.92 (6.45)	229.53 (45.64)	115.27 (40.03)	270.32 (55.75)	3.03 (1.10)
		Video-based	28.21 (6.23)	229.79 (53.60)	95.24 (30.21)	233.00 (52.30)	3.16 (1.01)
		Total	29.40 (6.26)	234.34 (50.99)	103.24 (35.57)	254.95 (64.55)	3.04 (1.02)
	Total(N=372)	Text	31.54 (6.38)	260.90 (57.65)	107.48 (34.08)	289.10 (83.33)	2.61 (1.09)
		Text-graphic composite	32.43 (6.29)	241.71 (44.71)	116.17 (37.32)	274.75 (61.20)	2.71 (1.12)
		Video-based	29.73 (6.15)	240.31 (59.56)	96.40 (28.24)	259.56 (73.71)	2.87 (1.13)
		Total	31.23 (6.36)	247.64 (55.04)	106.69 (34.30)	274.47 (74.10)	2.73 (1.12)

Supplementary Table 2 Pearson correlations between covariate measures and dependent variables in older knee arthroplasty patients undergoing preoperative digital health education (N = 372).

	Variables	Correlation	1	2	3	4	5	6	7	8	9	10	11
1	Knee function	r value	1.000										
		P value	--										
2	Prior knowledge	r value	0.186***	1.000									
		P value	<.001	--									
3	E-Health literacy	r value	-0.041	0.148**	1.000								
		P value	.43	.004	--								
4	Self-efficacy	r value	0.038	0.116*	-0.226**	1.000							
		P value	.47	.02	<.001	--							
5	Psychological distress	r value	-0.049	0.057	-0.187***	-0.073	1.000						
		P value	.35	.27	<.001	0.15	--						
6	Technophobia	r value	-0.192***	-0.019	-0.349***	-0.037	0.193***	1.000					
		P value	<.001	.72	<.001	.48	<.001	--					
7	NASA-TLX	r value	-0.091	-0.145**	-0.108*	-.162**	0.027	-0.003	1.000				
		P value	.08	.005	.03	.002	.61	.94	--				
8	Learning performance	r value	0.108*	0.114*	-0.002	.166**	-0.004	-0.071	-0.324***	1.000			
		P value	.03	.03	0.96	.001	.93	.17	<.001	--			
9	Average fixation duration	r value	-0.091	-0.065	0.003	-.133*	-0.046	-0.022	0.221***	-0.274***	1.000		
		P value	.08	.21	.96	.01	.37	.67	<.001	<.001	--		
10	Number of fixation points	r value	0.129*	0.065	-0.040	-.104*	0.003	-0.072	0.152**	0.000	0.024	1.00	
		P value	.01	.21	.44	.04	.95	.16	.003	.99	.64	--	
11	Duration before the first fixation	r value	-0.084	-0.066	-0.086	-.102*	0.056	0.056	0.153**	-0.154**	0.216***	0.126*	1.00
		P value	.10	.21	.09	0.05	.28	.28	.003	.003	<.001	.01	--

Note: **P* <.05, ***P* <.01, and ****P* <.001.

Supplement Table 3 Post Hoc of presentation modality on cognitive load and learning performance in older patients undergoing knee arthroplasty (N=372).

Dependent variable	Comparison of combinations	Mean difference	SE	*P* value [a]	95% CI	Cohen's d
NASA	Text vs Text-graphic composite	1.815	0.761	.05	-0.015 to 3.644	4.494
	Text vs Video-based	-0.887	0.761	.73	-2.716 to 0.942	-2.220
	Text-graphic composite vs Video-based	-2.702	0.761	.001	-4.531 to -0.873	-6.813
Average fixation duration	Text vs Text-graphic composite	20.589	6.72	.007	4.428 to 36.75	0.025
	Text vs Video-based	19.185	6.72	.01	3.025 to 35.346	0.023
	Text-graphic composite vs Video-based	-1.403	6.72	.99	-17.564 to 14.758	-0.002
Number of fixation points	Text vs Text-graphic composite	11.081	4.221	.03	0.929 to 21.232	0.020
	Text vs Video-based	-8.685	4.221	.12	-18.837 to 1.466	-0.017
	Text-graphic composite vs Video-based	-19.766	4.221	< .001	-29.917 to -9.615	-0.063
Duration before the first fixation	Text vs Text-graphic composite	29.54	8.914	.003	8.101 to 50.98	0.026
	Text vs Video-based	14.347	8.914	.32	-7.093 to 35.786	0.014
	Text-graphic composite vs Video-based	-15.194	8.914	.27	-36.633 to 6.246	-0.019
Learning performance	Text vs Text-graphic composite	-0.258	0.136	.18	-0.586 to 0.069	-0.015
	Text vs Video-based	-0.097	0.136	.99	-0.424 to 0.231	-0.006
	Text-graphic composite vs Video-based	0.161	0.136	.71	-0.166 to 0.489	0.009

a. Multiple comparison adjustment: Bonferroni method.

Supplement Table 4 Stratified analysis of the effects of presentation modality on cognitive load and learning performance by learning motivation level (high vs. low) in older patients undergoing knee arthroplasty (N=372).

Dependent variable	Independent variable	Comparison of combinations	Mean difference	SE	*t* test (df)	*P* value [a]	95% CI
NASA-TLX	Low learning motivation	Text vs Text-graphic composite	-1.94	1.08	1.799 (366)	.22	-4.534 to 0.654
		Text vs Video-based	1.76	1.08	1.632 (366)	.31	-0.834 to 4.354
		Text-graphic composite vs Video-based	3.70	1.08	3.43 (366)	.002	1.106 to 6.294
	High learning motivation	Text vs Text-graphic composite	0.16	1.08	0.1483 (366)	.99	-2.434 to 2.754
		Text vs Video-based	1.87	1.08	1.734 (366)	.25	-0.724 to 4.464
		Text-graphic composite vs Video-based	1.71	1.08	1.585 (366)	.34	-0.884 to 4.304
	Text	Low learning motivation vs High learning motivation	2.92	1.08	2.707 (366)	.007	0.799 to 5.041
	Text-graphic composite	Low learning motivation vs High learning motivation	5.02	1.08	4.654 (366)	< .001	2.899 to 7.141
	Video-based	Low learning motivation vs High learning motivation	3.03	1.08	2.809 (366)	.005	0.909 to 5.151
Average fixation duration	Low learning motivation	Text vs Text-graphic composite	24.19	9.49	2.548 (366)	.03	1.361 to 47.020
		Text vs Video-based	27.26	9.49	2.872 (366)	.01	4.431 to 50.090
		Text-graphic composite vs Video-based	3.07	9.49	0.3234 (366)	.99	-19.760 to 25.900
	High learning motivation	Text vs Text-graphic composite	14.18	9.49	1.494 (366)	.41	-8.649 to 37.010
		Text vs Video-based	13.92	9.49	1.467 (366)	.43	-8.909 to 36.750
		Text-graphic composite vs Video-based	-0.26	9.49	0.02739 (366)	.99	-23.090 to 22.570
	Text	Low learning motivation vs High learning motivation	34.37	9.49	3.621 (366)	< .001	15.700 to 53.040
	Text-graphic composite	Low learning motivation vs High learning motivation	24.36	9.49	2.566 (366)	.01	5.694 to 43.030
	Video-based	Low learning motivation vs High learning motivation	21.03	9.49	2.216 (366)	.03	2.364 to 39.700
Number of fixation points	Low learning motivation	Text vs Text-graphic composite	-1.30	5.96	0.218 (366)	.99	-15.640 to 13.040
		Text vs Video-based	18.20	5.96	3.052 (366)	.007	3.858 to 32.540
		Text-graphic composite vs Video-based	19.50	5.96	3.27 (366)	.004	5.158 to 33.840
	High learning motivation	Text vs Text-graphic composite	-16.06	5.96	2.693 (366)	.02	-30.400 to -1.718
		Text vs Video-based	3.97	5.96	0.6657 (366)	.99	-10.370 to 18.310
		Text-graphic composite vs Video-based	20.03	5.96	3.359 (366)	.003	5.688 to 34.370
	Text	Low learning motivation vs High learning motivation	16.55	5.96	2.775 (366)	.006	4.823 to 28.280
	Text-graphic composite	Low learning motivation vs High learning motivation	1.79	5.96	0.3002 (366)	.76	-9.937 to 13.520
	Video-based	Low learning motivation vs High learning motivation	2.32	5.96	0.389 (366)	.69	-9.407 to 14.050
Duration before the first fixation	Low learning motivation	Text vs Text-graphic composite	37.48	12.59	2.977 (366)	.009	7.196 to 67.760
		Text vs Video-based	30.55	12.59	2.426 (366)	.04	0.266 to 60.830
		Text-graphic composite vs Video-based	-6.93	12.59	0.5504 (366)	.99	-37.210 to 23.350
	High learning motivation	Text vs Text-graphic composite	-8.79	12.59	0.6981 (366)	.99	-39.070 to 21.490
		Text vs Video-based	28.53	12.59	2.266 (366)	.07	-1.754 to 58.810
		Text-graphic composite vs Video-based	37.32	12.59	2.964 (366)	.01	7.036 to 67.600
	Text	Low learning motivation vs High learning motivation	55.13	12.59	4.378 (366)	< .001	30.370 to 79.890
	Text-graphic composite	Low learning motivation vs High learning motivation	8.86	12.59	0.7036 (366)	.48	-15.900 to 33.620
	Video-based	Low learning motivation vs High learning motivation	53.11	12.59	4.218 (366)	< .001	28.350 to 77.870
Learning performance	Low learning motivation	Text vs Text-graphic composite	-0.10	0.19	0.5182 (366)	.99	-0.564 to 0.364
		Text vs Video-based	-0.29	0.19	1.503 (366)	.40	-0.754 to 0.174
		Text-graphic composite vs Video-based	-0.19	0.19	0.9845 (366)	.97	-0.654 to 0.274
	High learning motivation	Text vs Text-graphic composite	-0.09	0.19	0.4663 (366)	.99	-0.554 to 0.374
		Text vs Video-based	-0.22	0.19	1.14 (366)	.76	-0.684 to 0.244
		Text-graphic composite vs Video-based	-0.13	0.19	0.6736 (366)	.99	-0.594 to 0.334
	Text	Low learning motivation vs High learning motivation	-0.65	0.19	3.368 (366)	.001	-1.030 to -0.271
	Text-graphic composite	Low learning motivation vs High learning motivation	-0.64	0.19	3.316 (366)	.001	-1.020 to -0.261
	Video-based	Low learning motivation vs High learning motivation	-0.58	0.19	3.005 (366)	.003	-0.959 to -0.201

a. Multiple comparison adjustment: Bonferroni method.

Supplement Table 5 Pearson correlation analysis of cognitive load and learning performance across all participants in preoperative digital health education (N = 372).

Variable		NASA-TLX	Average fixation duration	Number of fixation points	Duration before the first fixation	Learning performance
NASA-TLX	r value	1.00				
	P value	--				
Average fixation duration	r value	.221***	1.00			
	P value	< .001	--			
Number of fixation points	r value	.152**	0.024	1.00		
	P value	.003	.64	--		
Duration before the first fixation	r value	.153**	.216**	.126*	1.00	
	P value	.003	0	.01	--	
Learning performance	r value	-.324***	-.274***	0	-.154**	1.00
	P value	< .001	< .001	.99	.003	--

Note: * P<.05; ** P <.01, *** P <.001

Supplementary File

Supplementary File 1

Health education materials presented in different forms

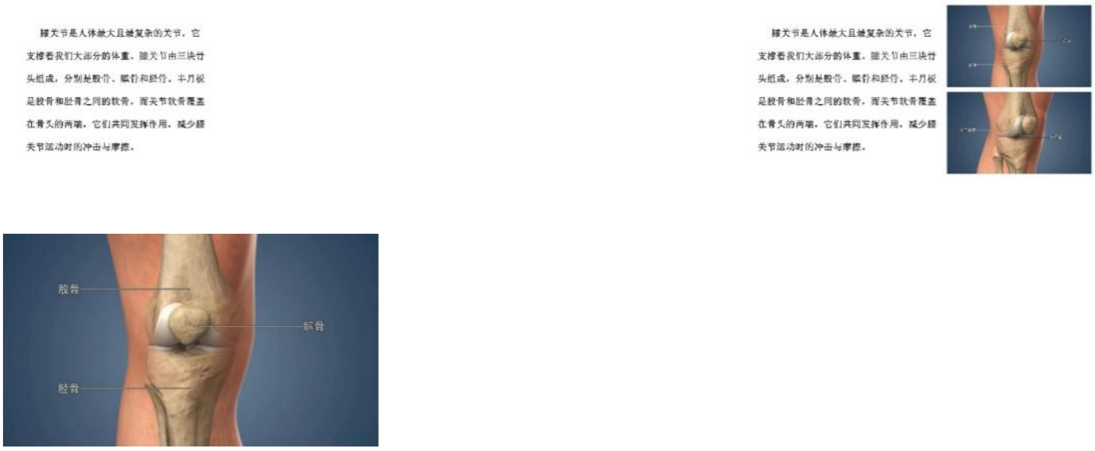

(a) knee anatomy

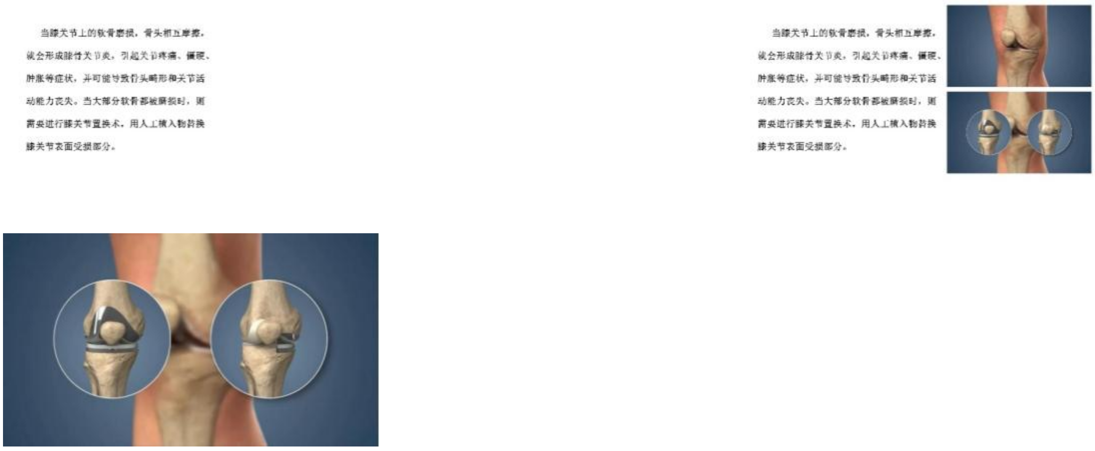

(b) procedure overview

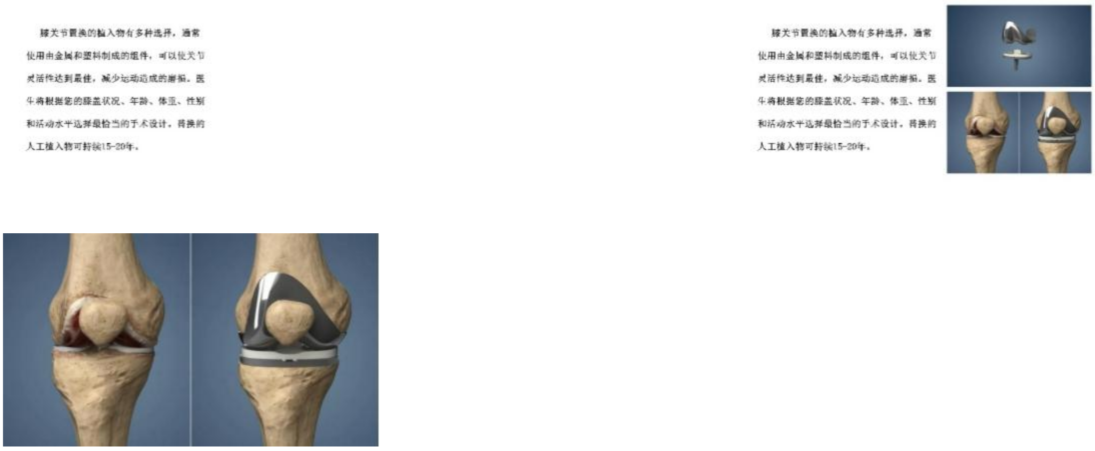

(c) implant selection

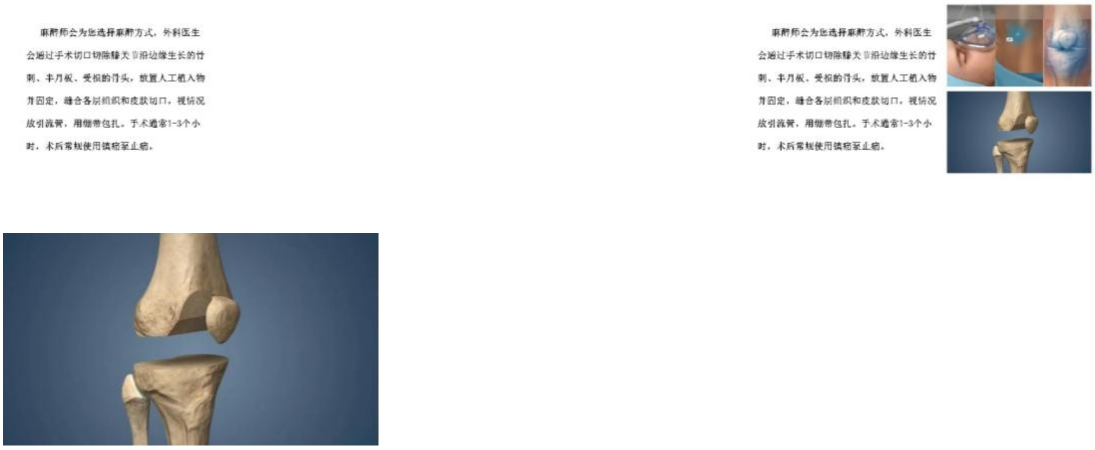

(d) surgical process

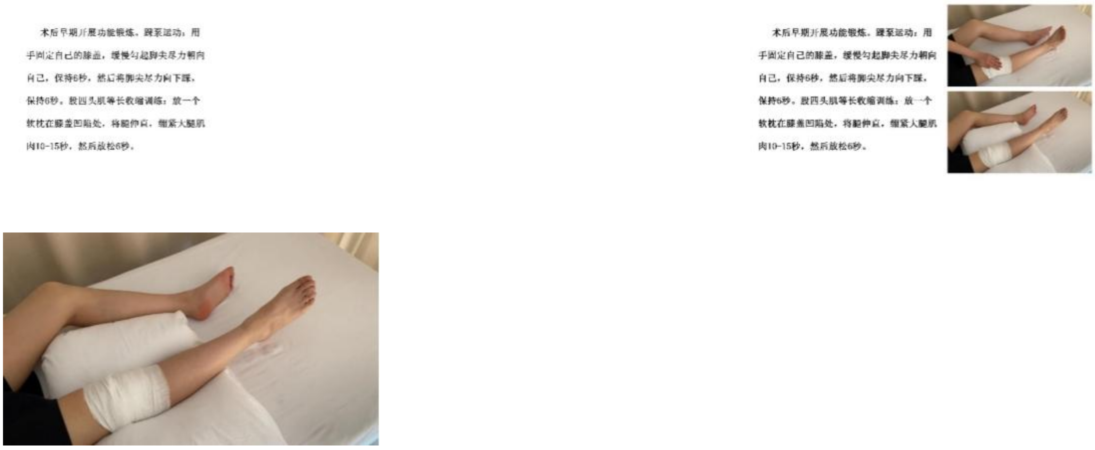

(e) rehabilitation exercises

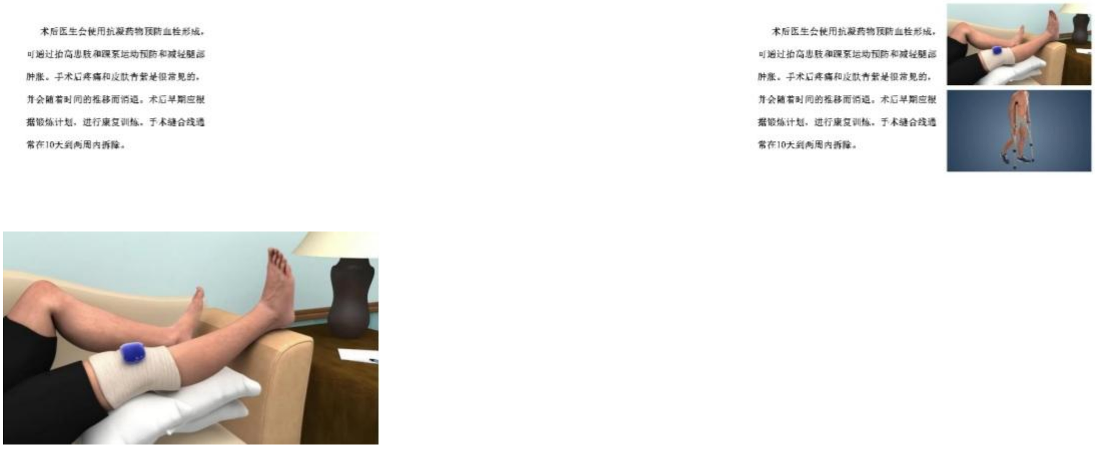

(f) expected outcomes

Supplementary File 2

**Sociodemographic characteristics**
**Gender:** 1. Male □ 2. Female □ **Age:**____ **Ethnicity:**____<br>**Religious Belief:** 1. Yes □ 2. No □<br>**Residence:** 1. Town □ 2. Rural □ 3. Urban □<br>**Marital Status:** 1. Married □ 2. Unmarried □ 3. Divorced □ 4. Widowed □<br>**Education Level:** 1. Illiterate □ 2. Primary School □ 3. Junior High School □<br>4. High School or Technical Secondary School □ 5. College or Above □<br>**Employment Status:** 1. Employed □ 2. Part-time □ 3. Self-employed □ 4. Retired □ 5. Unemployed □<br>**Occupation:** 1. Government Official □ 2. Public Institution Employee □ 3. Enterprise Employee □<br>4. Self-employed □ 5. Other □<br>**Average Monthly Household Income per Capita:** 1. ≤1000 □ 2. 1001-3000 □ 3. 3001-5000 □ 4. >5000 □<br>**Medical Payment Method:** 1. Public Expense □ 2. Medical Insurance □<br>3. Self-payment □ 4. New Rural Cooperative Medical Care □<br>**Have Children:** 1. Yes □ 2. No □<br>**Living Situation:** 1. Living with Spouse □ 2. Living with Children □ 3. Living Alone □<br>**Number of Children:** Care Type: 1. Family Care □ 2. Caregiver Care □ 3. No Care □<br>**Smoking History:** 1. Yes □ 2. No □<br>**Drinking History:** 1. Yes □ 2. No □<br>**Weight:**____kg **Height:**____cm **BMI:**____kg/m²

**Sociodemographic characteristics**
**Diagnosis:**____<br>**Disease Duration:**____years **WOMAC Osteoarthritis Index:**____points:<br>**Planned Surgery Date:**___year___month___day<br>**Planned Surgical Procedure:**____<br>**Planned Surgical Site:** 1. Left □ 2. Right □ 3. Bilateral □<br>1. Unicompartmental □ 2. Total Knee □<br>**Are there any other coexisting diseases?** 1. Yes □ 2. No □<br>**Coexisting Diseases: Diabetes:**□ Hypertension □ Stroke<br>□ Coronary Heart Disease □ Tumor □ Others:<br>**Medication History:**____

Supplementary File 3

Evaluation of Health Education Learning Performance

Instructions: This project is used to evaluate the effectiveness of your learning after digital health education. All questions below are single-choice questions.

Anatomy of the knee joint

The largest and most complex joint in the human body is ( )

A. Hip joint

B. Elbow joint

C. Shoulder joint

D. Knee joint

Grandpa Wang was diagnosed with severe knee osteoarthritis. The bone areas that may be worn down include ( )

A. Humerus, femur, metatarsal

B. Tibia, femur, patella

C. Tibia, fibula, patella

D. Humerus, fibula, metatarsal

What is the name of the cartilage between the femur and the tibia? ( )

A. Meniscus

B. Intervertebral disc

C. Ligament

D. Articular disc

Which of the following is NOT a function of joint cartilage? ( )

A. Reducing friction

B. Secreting synovial fluid

C. Facilitating movement

D. Cushioning shock

Which type of cartilage is most likely to be damaged in obese individuals? ( )

A. Ear cartilage

B. Costal cartilage

C. Meniscus

D. Intervertebral disc

Knee arthroplasty

How is knee osteoarthritis formed? ( )

A. Systemic infection

B. Cartilage wear

C. Nerve damage

D. Getting cold

Among the symptoms of knee osteoarthritis, which of the following is unlikely to occur? ( )
A. Pain
B. Stiffness
C. Pus formation
D. Swelling

In severe knee joint disease, which of the following is unlikely to occur? ( )
A. Bone deformity
B. Loss of mobility
C. Inability to bend the joint
D. Low back pain

Auntie Wang has knee osteoarthritis, and all conservative treatments have been ineffective. Which of the following treatments can be considered? ( )
A. Acupuncture and massage
B. Exercise training
C. Knee joint replacement surgery
D. Continue taking oral painkillers

Mr. Li often hears sounds in his joint(s) when walking. What might be the possible cause? ( )
A. Joint wear
B. Joint infection
C. Fracture
D. Sprain

Implant selection
Which of the following is NOT a common material for knee replacement implants? ( )
A. Metal
B. Ceramic
C. Bone
D. Plastic

What the doctor bases the choice of implant type and surgical method on does NOT include: ( )
A. Condition of the knee
B. Weight
C. Activity level
D. Diet

After knee replacement, under normal circumstances, the approximate lifespan of the prosthesis is: ( )
A. 10~15 years
B. 1~5 years
C. 5~10 years

D. 15~20 years

Auntie Wang plans to undergo knee replacement surgery. Which of the following is NOT an objective of the surgery? (　)

A. Improve joint mobility

B. Reduce pain

C. Correct deformity

D. Prevent infection

Uncle Li is preparing for knee replacement surgery. After admission, the doctor ordered an X-ray. The purpose does NOT include: (　)

A. Determine if the patient is obese

B. Assess the degree of knee joint wear

C. Select the implant

D. Choose the surgical plan

Surgical procedure

Who decides the type of anesthesia? (　)

A. The patient decides

B. The doctor decides

C. The anesthesiologist decides

D. The nurse decides

What part is replaced during knee replacement surgery? (　)

A. The damaged joint surface

B. The entire knee joint

C. The ends of the bones

D. The meniscus

After knee replacement surgery, which of the following is it unlikely you would bring back to the ward from the operating room? (　)

A. Bandages

B. Wound drain tube

C. Walker

D. Pain pump

Auntie Li is scheduled for knee replacement surgery at 9:00 AM. If everything goes smoothly, how long will it generally take before she returns to the ward? ( )

A. 9:30 AM

B. 2:00 PM

C. 11:30 AM

D. 3:00 PM

What is the purpose of bandaging? ( )

A. Protect the wound
B. Observe the wound condition
C. Reduce inflammation
D. Relieve pain

Rehabilitation exercises
What is the recommended time to start functional exercises after knee replacement surgery? ( )
A. After waking up from anesthesia
B. 3 days later
C. After the operated limb is completely pain-free
D. After discharge

Ms. Wang underwent knee replacement surgery. Which exercise should she start as soon as possible after the surgery? ( )
A. Ankle pump exercise
B. Walking
C. Getting out of bed
D. Climbing stairs

During the ankle pump exercise, how long should you try to point your toes up towards yourself? ( )
A. 3 seconds
B. 6 seconds
C. 10 seconds
D. 15 seconds

During quadriceps isometric contraction training, how long should you tighten your thigh muscles? ( )
A. 1-5 seconds
B. 5-10 seconds
C. 10-15 seconds
D. 15-30 seconds

In the early postoperative period, which exercise can be performed completely independently without assistance or tools? ( )
A. Getting out of bed and walking
B. Bending the knee
C. Quadriceps isometric contraction training
D. Ankle pump exercise

Postoperative outcomes
Which of the following is NOT a correct measure to prevent thrombosis after knee replacement surgery? ( )
A. Ankle pump exercise

B. Early mobilization out of bed
C. Bed rest
D. Using anticoagulant medication as prescribed by the doctor

What precautions should be taken after knee replacement surgery? (   )
A. Avoid repetitive strain
B. Perform rehabilitation exercises regularly according to the exercise plan
C. Keep the wound dry
D. All of the above

Sutures for the surgical incision in knee replacement surgery are usually removed within (   ) after the operation.
A. One week
B. Two weeks
C. One month
D. Two months

Regarding the functions of elevating the affected limb and performing ankle pump exercises, which statement is INCORRECT? ( )
A. Preventing thrombosis
B. Reducing swelling
C. Exercising the muscles
D. Reducing infection

Which of the following postoperative discomforts is INCORRECT? ( )
A. Skin bruising
B. Swelling
C. Pain
D. Drowsiness

Supplementary File 4

Instructions: This project is used to assess your prior knowledge. A score of 1 indicates no experience at all, while a score of 10 represents proficient use and benefit. Please mark "√" in the appropriate box on the right.

**Item**	1	2	3	4	5	6	7	8	9	10
1. Have you previously studied digital health education materials? (If yes, please describe the format and content of your study: ________________)										
2. Have you participated in traditional health education for knee joint diseases (e.g., face-to-face education, printed health education materials)?										
3. The following will test your knowledge of knee joint diseases:<br>(1) Do you know what the knee joint is composed of?										
(2) Do you know what knee osteoarthritis is?										
(3) Do you know the risk factors for knee osteoarthritis?										
(4) Do you know the treatment methods for knee osteoarthritis?										

Supplementary File 5

Illustration of AOIs (Taking the Anatomy of the Knee Joint as an Example)

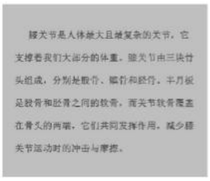

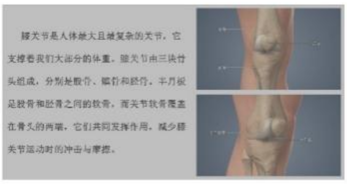

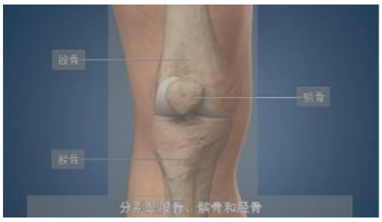

Supplement: Multimedia Appendix 1 [file jmir-v27-e79430-s001.pdf]
